# Analysis of miRNA expression profiles in the liver of *Clock*^Δ19^ mutant mice

**DOI:** 10.7717/peerj.8119

**Published:** 2019-11-28

**Authors:** Yanli Wang, Ke Lv, Mei Zhao, Hailong Chen, Guohua Ji, Yongliang Zhang, Tingmei Wang, Hongqing Cao, Yinghui Li, Lina Qu

**Affiliations:** 1 School of Life Sciences, Northwestern Polytechnical University, Xian, Shaanxi, China; 2 State Key Laboratory of Space Medicine Fundamentals and Application, China Astronaut Research and Training Center, Beijing, China; 3 Institute of Psychology, Chinese Academy of Sciences, Beijing, China

**Keywords:** Liver, Clock mutation, Circadian rhythms, miRNAs

## Abstract

The circadian clock controls the physiological functions of many tissues including the liver via an autoregulatory transcriptional−translational feedback loop, of which CLOCK is a core positive component. In addition, many studies have indicated that microRNAs (miRNAs) regulate liver function. However, how CLOCK-regulated miRNAs are linked to liver function remains largely unknown. In this study, miRNAs expression profiles were performed in the liver of *Clock*^Δ19^ mutant mice. Compared to wild type mice, totals of 61 and 57 putative CLOCK-regulated miRNAs were differentially expressed (fold change absolute value ≥2) at zeitgeber time 2 and zeitgeber time 14, respectively. According to the pathway analyses, the target genes of differentially expressed miRNAs were mainly involved in pathways in cancer, the PI3K-Akt signaling pathway and the MAPK signaling pathway. Protein−protein interaction analyses revealed that the hub genes were primarily associated with pathway in cancer and circadian rhythms. Expression validation showed that while the expression levels of miR-195 and miR-340 were up-regulated, the rhythms of these two miRNAs were always maintained. The expression level of nr1d2 mRNA was down-regulated. We identified a number of prospective CLOCK-regulated miRNAs that play roles in the various physiological processes of the liver, providing a reference to better understanding the potential regulatory mechanisms in the liver.

## Introduction

The circadian clock is an internal time keeping system that allows organisms to adapt physiological and behavioral processes to environmental light/dark cycles (Kinoshita et al., 2014). In mammals, the master clock is located in the suprachiasmatic nucleus (SCN) (Enoki et al., 2017). The SCN exhibits endogenous rhythms and controls circadian rhythms in peripheral tissues such as liver, heart and kidney (Menaker, Murphy & Sellix, 2013). In addition, to receiving the information transmitted by neuronal and humoral pathways from the SCN, the peripheral tissues also have an internal timing system, and several clock genes such as transcriptional activators (e.g., *Clock* and *Bmal1*) and repressors (e.g., *Per1* and *Per2*) form a transcriptional−translational feedback loop (Zhou et al., 2016). The core clock transcription factor, CLOCK, coupled with BMAL1 activates the transcription of repressors as well as clock-controlled genes (CCGs). There is one additional regulatory feedback loop, in which the stabilizing feedback loop relies on alternate repression by REV-ERBα and activation by RORα (Lee & Kim, 2013; Ripperger & Albrecht, 2012).

The liver is currently the most extensively studied peripheral tissue with respect to circadian rhythms. Recent studies have suggested that CLOCK contribute to a wide variety of physiological processes in the liver, such as cell proliferation (Miller et al., 2007), cell division (Matsuo et al., 2003), and obesity (Marcheva et al., 2010; Turek et al., 2005). Matsuo et al. (2003) found that, several key genes involved in cell-cycle progression, including *Wee1* and *Cdc2*, were direct targets of the CLOCK–BMAL1 complex. Oishi et al. (2003) revealed that CLOCK was involved in the transcriptional regulation of many circadian output genes, based on microarray analyses using liver RNA isolated from *Clock* mutant mice. However, those studies were mainly focused on the protein-encoding mouse transcriptome. It is necessary to identify a more complete cohort of genes that are post-transcriptionally regulated by CLOCK to fully understand the molecular mechanisms by which clock genes affect physiological processes in the liver.

MicroRNAs (miRNAs), short non-coding RNA molecules, can regulate gene expression by repressing gene transcription by binding to the 3′ untranslated region (3′UTR) of target mRNAs (Lai, 2002). Several studies have demonstrated that circadian miRNAs, which have been identified by microarray-based expression profiling or miRNA-sequencing (miR-seq), are involved in metabolic homeostasis and cell cycles (Na et al., 2009; Vollmers et al., 2012).

In the present study, we performed a microarray analyses of miRNAs in the liver of *Clock*^Δ19^ mutant mice to identify putative CLOCK-regulated miRNAs. Then we conducted bioinformatic analyses to identify the target genes of differentially expressed miRNAs and analyzed the pathways in which they are involved. Our results provide a unique basis for further unraveling of the role of putative CLOCK-regulated miRNAs in the liver.

## Materials and Methods

### Ethics statement

All animal procedures used in this study were conducted in compliance with animal protection protocol as approved by the Institutional Animal Care and Use Committee in China. All the experimental procedures were approved by the Committees of Animal Ethics and Experimental Safety of China Astronaut Research and Training Center, and the ethical approval number was “2012-045.”

### Animals

*Clock* mutant mice (*Clock*^Δ19^) were acquired from the Institute of Psychology, Chinese Academy of Sciences, Beijing, China. All male mice at 6−8 weeks of age used in this study were individually caged and maintained under IVC conditions with 12 h light/dark cycle, lights-on at 07:00 h and free access to food and water for 2 weeks before using. At 09:00 h zeitgeber time 2 (ZT2), 21:00 h zeitgeber time 14 (ZT14) on the first day after 2 weeks adaptation period, three animals of each group were sacrificed by dry ice. The liver was isolated, quickly frozen and stored in liquid nitrogen. For analyzing genes expression by real-time PCR, *Clock* mutant mice and their wild type controls (5 mice of each group per time point) were killed every 4 h across 26 h at 09:00 h (ZT2), 13:00 h (ZT6), 17:00 h (ZT10), 21:00 h (ZT14), 01:00 h (ZT18), 05:00 h (ZT22) and 09:00 h of the following day (ZT2).

### Total RNA isolation and microarray hybridization

According to illumina’s protocol, the three RNA samples at each time point were mixed and isolated using the mirVana miRNA extraction Kit (Ambion, Austin, TX, USA). RNA labeling, microarray hybridization and data processing were analyzed by Shanghai OE Biotech. Co. Ltd., Briefly, RNA labeled and hybridized using the Agilent mouse 4 × 44 K microRNA microarray. Hybridization signals were detected with an Agilent DNA microarray scanner G2565BA, and scan images were analyzed using Agilent feature extraction software. Data is analyzed using Agi4×44PreProcess (Agilent Technologies, Santa Clara, CA, USA). The Fold Change Absolute larger than or equal to two was considered to have an evident difference.

### MicroRNA target prediction and functional annotation

The predicted targets of miRNAs (both changed at two time points with FCA ≥ 2) were analyzed by miRwalk (Dweep & Gretz, 2015), the predicted targets obtained from at least four programs in miRanda, miRDB, RNAhybrid, PICTAR 5, PITA and Targetscan were regarded as the putative target genes. Then the putative target genes were integrated into David (DAVID 6.7; https://david-d.ncifcrf.gov/) to analyze Gene Ontology and the Kyoto Encyclopedia of Genes and Genomes (KEGG) pathway (Huang, Sherman & Lempicki, 2009a, 2009b). Based on the interactions of miRNAs and target genes, the miRNA function network was built.

### Integration of protein–protein interaction network and module analysis

To evaluate the interactive relationships among prediction target genes, all genes were mapped to an online tool, STRING (Version 10.0) (Szklarczyk et al., 2015), to evaluate the Protein−protein interaction (PPI) information. The experimentally validated interactions with a combined score >0.4 were considered significant. Then, the Molecular Complex Detection (MCODE), one plug-in unit of Cytoscape (Shannon et al., 2003) was utilized to screen the modules of PPI network. The criteria were: for MCODE scores >5 and number of nodes >10. Moreover, the function and pathway enrichment analysis of the genes in the modules were performed. *p* < 0.05 was regarded as significant differences.

### Real time quantitative polymerase chain reaction

To validate the expression of miRNAs of interest, total RNA was extracted from liver tissue using the TRIzol regent (Cat. #15596026; Invitrogen, Carlsbad, CA, USA) according to the manufacturer’s instructions. Then the RNA was reversed and poly A tailed using PrimeScript™ miRNA qPCR starter kit ver. 2.0 (Cat. # RR718; TaKaRa, Shiga, Japan). The cDNA was used as the template for the qPCR, which was performed using the SYBR^®^ Premix Ex Taq™II (Cat. #DRR820A; TaKaRa, Shiga, Japan).The sequences of primers used for qRT-PCR were presented in Table 1. Amplification data were analyzed using the comparative threshold (2^−ΔΔCt^) method after normalization to U6.

**Table 1:** The primers used for qRT-PCR analysis.

Gene	Forward sequences (5′–3′)	Reverse sequences (5′–3′)
U6	CTCGCTTCGGCAGCACATATACT	ACGCTTCACGAATTTGCGTGTC
miR-195	CGTAGCAGCACAGAAATATTGGC	
miR-338	AACAATATCCTGGTGCTGAGTG	
miR-340	CGGTCCGTCTCAGTTACTTTATAG	
miR-374	CGGATATAATACAACCTGCTAAGTG	
miR-669d	ACTTGTGTGTGCATGTATATGT	

**Note:**

The reverse primer of miRNAs referred to the manual of Prime Script™ miRNA qPCR starter Kit ver. 2.0 (Cat. #RR718; TaKaRa, Shiga, Japan).

## Results

### Identification of differentially expressed miRNA

**Figure 1:**
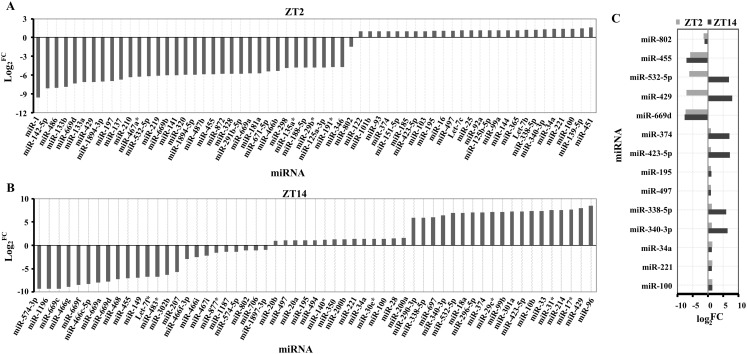
Differentially expressed miRNAs in the liver of *Clock*^Δ19^ mutant mice. (A) miRNAs changed in clock mutant mice compared with WT mice at ZT2. (B) miRNAs changed in *Clock* mutant mice compared with WT mice at ZT14. (C) miRNAs altered both in ZT2 and ZT14 under *Clock* mutant conditions. FC was the meaning of Fold Change. The value exceeds and below zero were considered up and down, respectively. All selected miRNAs were FCA ≥ 2.

A total of 194 miRNAs were identified in the liver of *Clock* mutant mice compared to WT mice (Table S1) at ZT2, of which the expression of 61 miRNAs (35 downregulated miRNAs and 26 upregulated miRNAs) differed by at least 1 fold (log_2_^fold change^ ≥ 1) (Fig. 1A). Among 157 miRNAs (Table S2), 24 downregulated and 33 upregulated miRNAs were differentially expressed between *Clock* mutant and control mice at ZT14 (Fig. 1B). Of these miRNAs, miR-100, miR-221, miR-34a, miR-340-3p, miR-338-5p, miR-497, miR-195, miR-423-5p, miR-374, miR-669d, miR-429, miR-532-5p, miR-455 and miR-802, were differentially expressed at both time points (Fig. 1C). Next, these 14 conserved miRNAs were evaluated by downstream target prediction and biological annotation enrichment analyses.

### miRNA regulatory network

**Figure 2:**
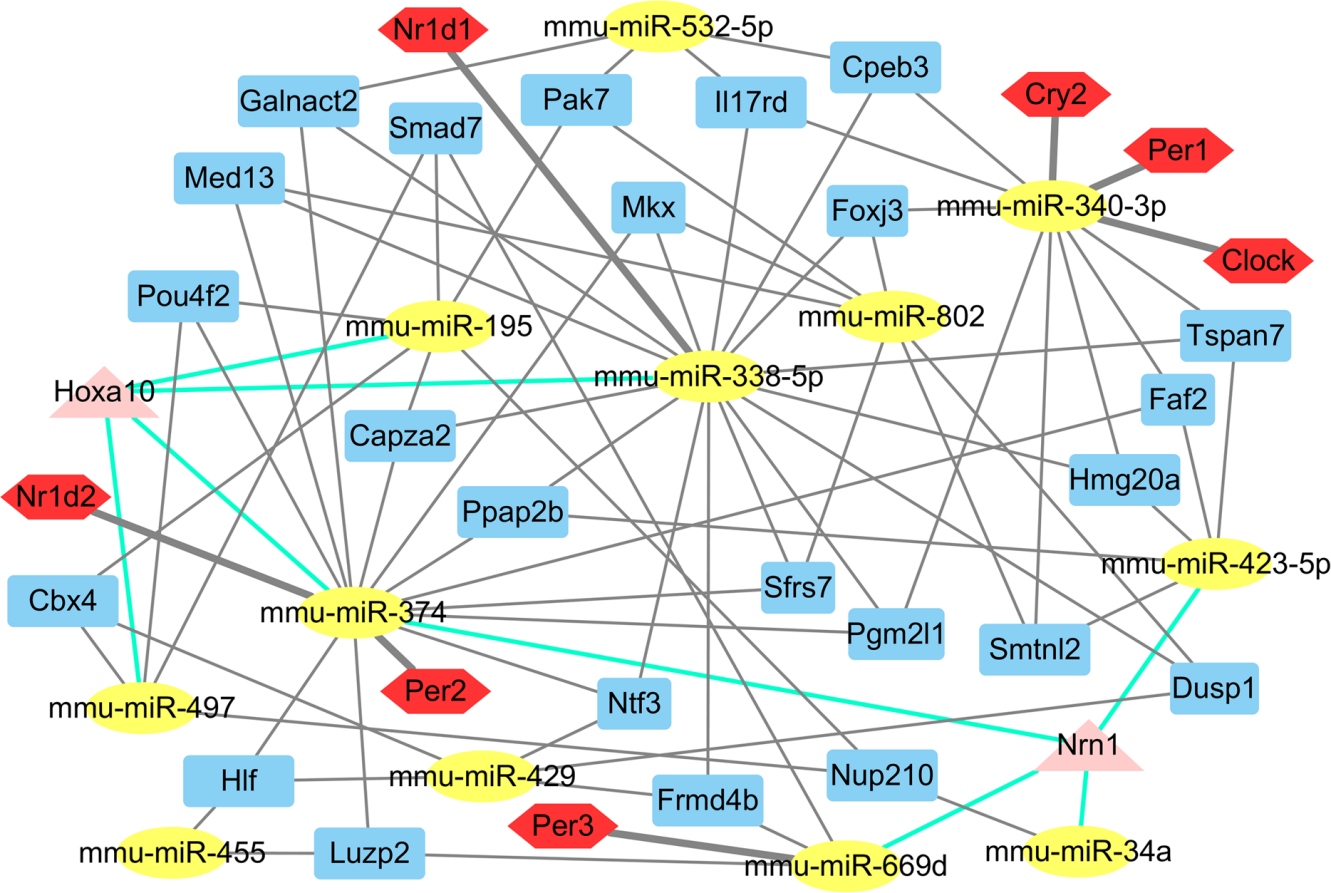
The main miRNA-target mRNA regulatory network of 14 miRNAs. The yellow ellipses indicate miRNAs, others are genes. Red hexagons represent circadian genes. Blue rectangles represent the genes, which are controlled by three miRNAs; triangles in pink indicate the genes, which are controlled by four miRNAs.

The putative target genes of the 14 differentially expressed miRNAs were predicted by miRwalk to obtain a more comprehensive understanding of their roles in the liver. Of these 1,781 putative miRNA-mRNA pairs, several core circadian clock gene-miRNA pairs (miR-340-3p targeting *Clock*, *Per1*, *Cry2*; miR-669d targeting *Per2*; miR-374 targeting *Per3* and miR-338-5p targeting *Nr1d1*) were identified (Table S3; Fig. 2). Twenty four genes were identified as potential targets of three miRNAs. Notably, the Nrn1 gene was regulated by four miRNAs: miR-34a, miR-374, miR-423-5p, and miR-669d. In addition, miR-195, miR-338-5p, miR-374 and miR-497 together regulate the *Hoxa10* gene (Fig. 2).

### Gene ontology and KEGG pathway analyses of the differentially expressed miRNA targets

**Figure 3:**
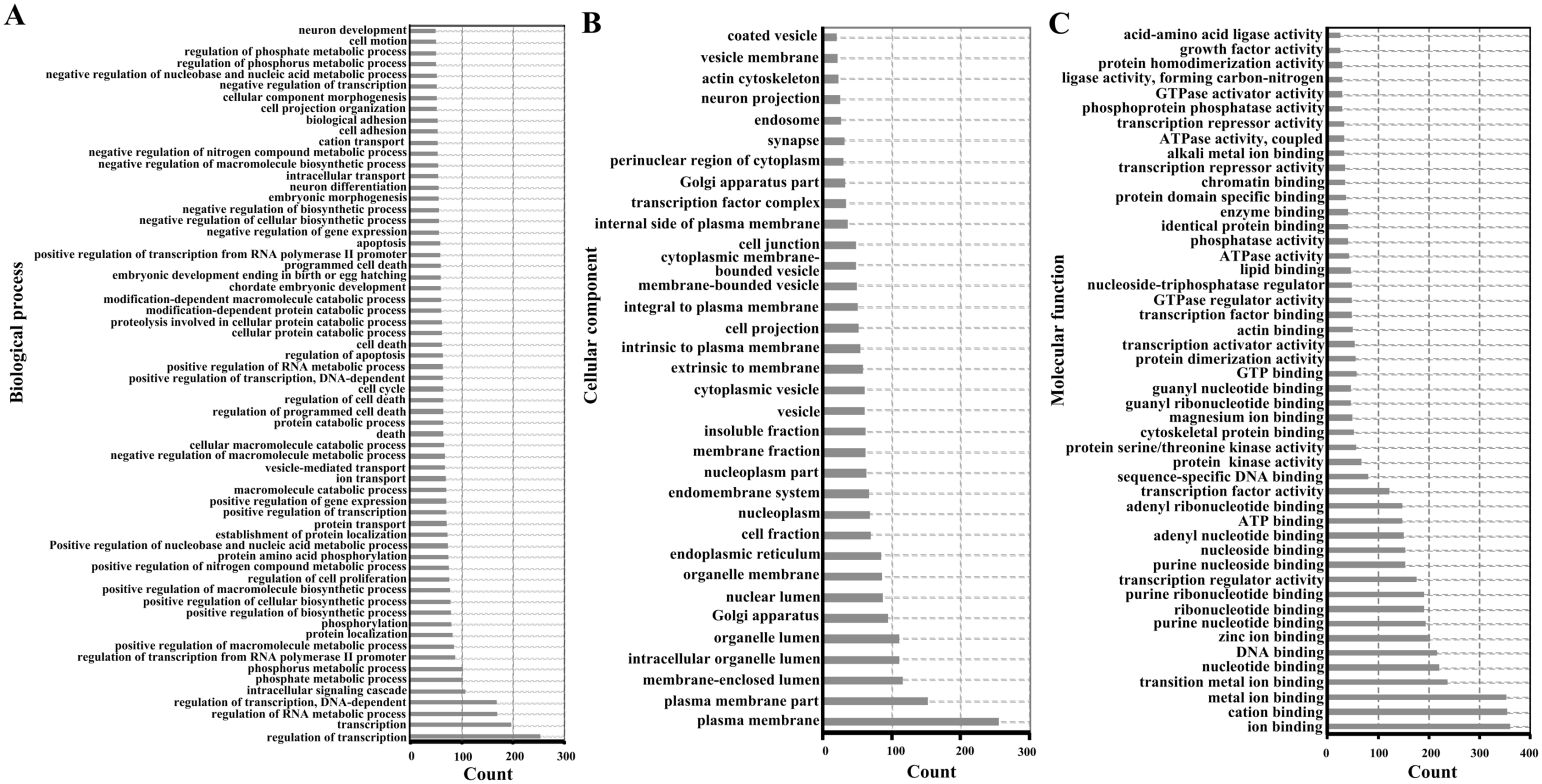
Enriched gene ontology terms of predicted targets of 14 differentially expressed miRNAs in the liver of *Clock* mutant mice. (A) Biological process. (B) Cellular component. (C) Molecular function.

For functional enrichment analyses of miRNAs, we uploaded the full list of target genes to DAVID 6.7 and extracted clustering information of the biological process (BP), cellular component (CC), molecular function (MF), and KEGG pathway categories. The BP category were mostly involved in regulation of transcription, regulation of RNA metabolic process, and intracellular signaling cascade (Fig. 3A). As shown in Fig. 3B, CC category analyses revealed that the target genes were highly associated with plasma membrane, membrane-enclosed lumen, and intracellular organelle lumen. In the MF category, the three most enriched terms were ion binding, cation binding and metal ion binding (Fig. 3C). The three most enriched KEGG pathways were the pathways in cancer, PI3K-Akt signaling pathway, and the MAPK signaling pathway (Table 2).

**Table 2:** The KEGG pathway of the potential target genes. The KEGG pathway were analyzed by the predicted target genes of 14 differentially expressed miRNAs.

Term	Count	*p* Value	Genes
Pathways in cancer	54	4.13E−05	E2F2, ADCY1, FGF7, PPARG, LPAR4, FOXO1, LPAR1, PTEN, CCNE1, MAX, FOS, RALA, PLCB1, AKT3, PTGER4, ROCK2, RUNX1T1, LEF1, STK4, VEGFC, EP300, CRKL, HIF1A, GNB2, GNAQ, JUN, MAPK3, MAPK9, GNAS, WNT5A, GNAI3, BCL2L1, TCF7L2, IGF1R, RAC1, RUNX1, TRAF3, AXIN1, COL4A3, BMP2, MAP2K1, CBL, SMAD3, RAF1, HGF, FZD5, KITL, FZD4, FZD7, NRAS, CBLB, CDKN1A, LAMA5, GNG10
PI3K-Akt signaling pathway	42	0.004263	CRTC2, FGF7, PPP2R5A, CSF1, LPAR4, BCL2L1, LPAR1, PTEN, CCNE1, IGF1R, COMP, PPP2CA, RAC1, CREB3L1, ANGPT1, PDGFD, COL11A1, INSR, PPP2R2D, AKT3, COL4A3, SGK1, MAP2K1, ITGA1, RAF1, HGF, ITGA4, KITL, COL5A2, IL6RA, NRAS, VEGFC, CDKN1A, GNB2, CCND2, LAMA5, GNG10, MAPK3, YWHAQ, EFNA4, COL24A1, IL2
MAPK signaling pathway	37	2.07E−04	IL1R1, FGF7, TNF, MRAS, DUSP10, CACNB2, CACNB4, MAX, FOS, MAP3K2, MAP3K1, RAC1, MAP3K8, PPP3CA, MAP2K6, AKT3, NFATC1, NTF3, MAP2K1, TAOK1, NF1, MAP2K4, RAF1, STK4, NRAS, CRKL, MAPK12, DUSP1, RPS6KA2, JUN, MAPK3, MAPK9, RAP1B, DUSP8, GADD45A, CACNA1D, DUSP6
HTLV-I infection	35	0.004332	WNT5A, CRTC2, E2F2, IL1R1, ADCY1, TNF, MRAS, ITGB2, BCL2L1, MYBL2, MSX2, FOS, MAP3K1, PPP3CA, AKT3, TBPL1, NFATC1, KAT2B, MAP2K4, SMAD3, FZD5, FZD4, FZD7, NRAS, CDKN1A, SLC25A31, MSX1, ATF3, EP300, MAD2L1, CCND2, ETS1, JUN, ETS2, IL2
Proteoglycans in cancer	34	2.73E-05	WNT5A, CAV2, CAV1, TNF, MRAS, CAMK2G, LUM, SDC4, HOXD10, IQGAP1, SDC2, IGF1R, ANK2, RAC1, FRS2, CAMK2A, AKT3, MAP2K1, ROCK2, CBL, RAF1, HGF, FZD5, DDX5, FZD4, PPP1CB, FZD7, NRAS, CDKN1A, CBLB, HIF1A, MAPK12, MAPK3, HBEGF
Focal adhesion	34	4.10E−05	CAV2, CAV1, PTEN, VCL, IGF1R, PAK7, ARHGAP5, PAK4, COMP, RAC1, PDGFD, COL11A1, AKT3, COL4A3, VAV3, ACTN4, MAP2K1, ROCK2, ITGA1, RAF1, ACTN1, HGF, ITGA4, COL5A2, PPP1CB, VEGFC, CRKL, CCND2, LAMA5, JUN, MAPK3, MAPK9, RAP1B, COL24A1
Rap1 signaling pathway	33	1.86E−04	ADCY1, FGF7, GNAI3, CSF1, MRAS, LPAR4, ITGB2, LPAR1, IGF1R, CNR1, RAC1, RALA, ANGPT1, PDGFD, PLCB1, INSR, AKT3, MAP2K6, MAP2K1, RAF1, HGF, KITL, DOCK4, NRAS, VEGFC, CRKL, GNAQ, MAPK12, ID1, MAPK3, GNAS, RAP1B, EFNA4
Ras signaling pathway	31	0.002606	FGF7, CSF1, MRAS, ARF6, BCL2L1, IGF1R, PAK7, PAK4, RAC1, RALA, ANGPT1, PDGFD, INSR, AKT3, MAP2K1, NF1, RAF1, HGF, STK4, KITL, NRAS, VEGFC, GNB2, ETS1, GNG10, ETS2, MAPK3, MAPK9, PLA2G6, RAP1B, EFNA4
cAMP signaling pathway	30	4.80E−04	ADCY1, GNAI3, CAMK2G, PDE3B, SOX9, FOS, RAC1, PDE4B, CREB3L1, SUCNR1, CAMK2A, AKT3, NFATC1, VAV3, MAP2K1, ROCK2, HTR4, RAF1, CFTR, GRIA4, ATP1A2, PPP1CB, EP300, GRIA2, JUN, MAPK3, MAPK9, GNAS, RAP1B, CACNA1D
Endocytosis	29	0.082734	CAV2, CAV1, CHMP4B, CAPZA2, SNX2, ARF6, SNX4, EEA1, CLTC, AMPH, IGF1R, ARPC2, SH3GLB1, WWP1, SPG20, DNAJC6, VPS4A, WIPF1, IQSEC2, CBL, KIF5C, PSD3, SMAD3, A230046K03RIK, ARPC1A, CBLB, RAB35, WASL, RAB10
Oxytocin signaling pathway	28	6.06E−05	ADCY1, GNAI3, CAMK2G, CACNB2, CACNB4, KCNJ12, KCNJ14, FOS, PPP3CA, PLCB1, CAMK2A, NFATC1, MAP2K1, ROCK2, PRKAB2, PRKAB1, RAF1, PPP1CB, KCNJ5, CD38, NRAS, CDKN1A, GNAQ, JUN, MAPK3, GNAS, GUCY1B3, CACNA1D
Regulation of actin cytoskeleton	27	0.012774	FGF7, MRAS, IQGAP3, ITGB2, IQGAP1, VCL, PAK7, ARPC2, PAK4, RAC1, PDGFD, VAV3, MAP2K1, ACTN4, ROCK2, ITGA1, ACTN1, RAF1, ITGA4, PPP1CB, ARPC1A, NRAS, CRKL, MAPK3, CYFIP1, WASL, PIP4K2B
Dopaminergic synapse	26	2.50E−05	GNAI3, PPP2R5A, CAMK2G, KIF5C, MAOB, GRIA4, PPP1CB, KCNJ5, FOS, GNAQ, GRIA2, GNB2, MAPK12, PPP2CA, GNG10, SLC18A2, CREB3L1, MAPK9, GNAS, PPP3CA, PLCB1, CACNA1D, CAMK2A, CLOCK, AKT3, PPP2R2D
FoxO signaling pathway	26	2.50E−05	SGK1, MAP2K1, PRKAB2, PRKAB1, FOXO1, SMAD3, RAF1, HOMER1, SIRT1, STK4, PTEN, IL10, NRAS, IGF1R, TNFSF10, PLK4, CDKN1A, EP300, MAPK12, CCND2, MAPK3, MAPK9, KLF2, GADD45A, INSR, AKT3
Signaling pathways regulating pluripotency of stem cells	26	4.19E−05	WNT5A, BMI1, MEIS1, ACVR1C, ACVR1B, IGF1R, AKT3, AXIN1, BMP2, MAP2K1, MYF5, SMAD5, SMAD3, RAF1, FZD5, FZD4, FZD7, NRAS, ACVR2A, MAPK12, ID1, MAPK3, ID4, BMPR1B, ZFHX3, BMPR1A
Axon guidance	25	3.80E−05	GNAI3, PLXNA2, EPHB2, SEMA5A, PAK7, UNC5B, SEMA3F, PAK4, SEMA3E, RAC1, PPP3CA, SEMA3A, LRRC4, ROCK2, NTNG1, SLIT2, SLIT3, NCK2, EPHA4, SEMA6A, NRAS, EPHA7, RND1, MAPK3, EFNA4
Hippo signaling pathway	25	4.73E−04	WNT5A, BTRC, GDF6, ITGB2, TCF7L2, LATS2, CTGF, PPP2CA, DLG3, FBXW11, PPP2R2D, AXIN1, BMP2, SMAD3, LEF1, FZD5, SNAI2, FZD4, PPP1CB, FZD7, ID1, CCND2, YWHAQ, BMPR1B, BMPR1A
Wnt signaling pathway	24	4.22E−04	WNT5A, ROCK2, CAMK2G, BTRC, LEF1, FZD5, DAAM2, TCF7L2, FZD4, FZD7, EP300, SFRP1, CCND2, JUN, RAC1, MAPK9, WIF1, PPP3CA, RUVBL1, PLCB1, CAMK2A, FBXW11, AXIN1, NFATC1
Transcriptional misregulation in cancer	24	0.003952	BMI1, CCNT2, NFKBIZ, LDB1, PPARG, RUNX1T1, FOXO1, TSPAN7, BCL2L1, AFF1, DDX5, MEIS1, MYCN, IGF1R, MAX, CDKN1A, FLI1, SP1, CCND2, GOLPH3, ETV6, RUNX1, HPGD, MLLT3
TGF-beta signaling pathway	23	2.58E−07	BMP2, TNF, LTBP1, E2F5, SMAD7, GDF6, SMAD6, SMAD5, SMAD3, ACVR1C, ACVR2A, ACVR1B, EP300, SP1, ID1, PPP2CA, MAPK3, TGIF1, ID4, BMPR1B, PITX2, BMPR1A, TFDP1
cGMP-PKG signaling pathway	23	0.011286	ADCY1, GNAI3, MAP2K1, ROCK2, RAF1, PDE3B, ATP1A2, PRKG1, PPP1CB, KCNMB2, MEF2D, SLC25A31, GNAQ, MAPK3, ADRA1B, CREB3L1, GUCY1B3, PPP3CA, PLCB1, INSR, CACNA1D, AKT3, NFATC1
Adrenergic signaling in cardiomyocytes	22	0.005094	ADCY1, GNAI3, PPP2R5A, CAMK2G, CACNB2, ATP1A2, CACNB4, PPP1CB, TPM1, GNAQ, MAPK12, PPP2CA, MAPK3, KCNE1, ADRA1B, CREB3L1, GNAS, PLCB1, CACNA1D, CAMK2A, AKT3, PPP2R2D
Protein processing in endoplasmic reticulum	22	0.017745	SEC23A, RAD23B, SEC24A, DERL1, SYVN1, UBE4B, RNF185, DNAJB12, STUB1, SSR1, UBE2E3, BAK1, HSPA4L, ERN1, SIL1, MAPK9, AMFR, SEC24C, SEC24D, SAR1A, SEL1L, DNAJA2
T cell receptor signaling pathway	21	1.03E−04	VAV3, TNF, MAP2K1, CBL, CTLA4, RAF1, IL10, NCK2, NRAS, FOS, PAK7, CBLB, MAPK12, JUN, PAK4, MAPK3, MAP3K8, PPP3CA, AKT3, IL2, NFATC1
Insulin signaling pathway	21	0.004921	MAP2K1, CBL, PRKAB2, PHKA1, PRKAB1, ACACA, FOXO1, PDE3B, RAF1, PPP1CB, NRAS, PPP1R3D, CBLB, CRKL, PPP1R3F, MAPK3, FASN, MAPK9, PTPN1, INSR, AKT3
GnRH signaling pathway	20	2.91E−05	ADCY1, MAP2K1, CAMK2G, MAP2K4, RAF1, PRKCD, NRAS, GNAQ, MAPK12, MAP3K2, JUN, MAP3K1, MAPK3, MAPK9, HBEGF, GNAS, PLCB1, CACNA1D, CAMK2A, MAP2K6
Circadian entrainment	20	1.37E−04	ADCY1, GNAI3, CAMK2G, GRIA4, PRKG1, KCNJ5, FOS, GNAQ, GRIA2, GNB2, GNG10, MAPK3, PER2, PER1, GNAS, GUCY1B3, PER3, PLCB1, CACNA1D, CAMK2A
Melanogenesis	20	1.57E−04	WNT5A, ADCY1, GNAI3, MAP2K1, CAMK2G, LEF1, RAF1, FZD5, KITL, FZD4, TCF7L2, FZD7, NRAS, EP300, GNAQ, MAPK3, CREB3L1, GNAS, PLCB1, CAMK2A
Sphingolipid signaling pathway	20	0.002751	GNAI3, TNF, SPTLC1, MAP2K1, PPP2R5A, ROCK2, RAF1, SGMS1, PTEN, NRAS, S1PR3, GNAQ, MAPK12, PPP2CA, RAC1, MAPK3, MAPK9, PLCB1, AKT3, PPP2R2D
Hepatitis B	20	0.015866	E2F2, TNF, MAP2K1, MAP2K4, RAF1, PTEN, DDX58, NRAS, FOS, CCNE1, CDKN1A, EP300, JUN, MAP3K1, MAPK3, YWHAQ, CREB3L1, MAPK9, AKT3, NFATC1
ErbB signaling pathway	19	8.50E−05	MAP2K1, CAMK2G, CBL, MAP2K4, RAF1, NCK2, NRAS, PAK7, CBLB, CDKN1A, CRKL, EREG, JUN, PAK4, MAPK3, HBEGF, MAPK9, CAMK2A, AKT3
Chagas disease (American trypanosomiasis)	19	7.62E−04	ADCY1, TNF, GNAI3, MAP2K4, SMAD3, IL10, FOS, GNAQ, MAPK12, JUN, PPP2CA, MAPK3, IL12A, MAPK9, GNAS, PLCB1, AKT3, PPP2R2D, IL2
Retrograde endocannabinoid signaling	19	7.62E−04	GABRG1, ADCY1, GABRA2, GNAI3, GABRA1, GRIA4, KCNJ5, SLC17A6, NAPEPLD, GNAQ, GRIA2, GNB2, MAPK12, CNR1, GNG10, MAPK3, MAPK9, PLCB1, CACNA1D
Cholinergic synapse	19	0.002276	ADCY1, GNAI3, MAP2K1, CAMK2G, KCNJ12, KCNJ14, NRAS, FOS, GNAQ, GNB2, GNG10, MAPK3, CHRNA4, CREB3L1, SLC5A7, PLCB1, CACNA1D, CAMK2A, AKT3
Neurotrophin signaling pathway	19	0.005293	MAP2K1, NTF3, CAMK2G, RAF1, PRKCD, NRAS, CRKL, MAPK12, PRDM4, RPS6KA2, JUN, MAP3K1, RAC1, MAPK3, MAPK9, RAP1B, FRS2, CAMK2A, AKT3
Lysosome	19	0.005293	AGA, CTSZ, AP4E1, AP1B1, CLTC, GNS, NPC1, AP1S2, LAPTM5, LAMP3, GNPTAB, AP3M1, AP3B2, GALC, GAA, AP3D1, GGA2, CLN5, AP3B1
AMPK signaling pathway	19	0.008048	SCD1, CRTC2, PPP2R5A, PPARG, PRKAB2, PRKAB1, ACACA, FOXO1, CFTR, SIRT1, ADIPOQ, IGF1R, PPP2CA, FASN, CREB3L1, RAB10, INSR, AKT3, PPP2R2D
Vascular smooth muscle contraction	19	0.008048	ADCY1, MAP2K1, ROCK2, CALD1, PRKCH, RAF1, PRKG1, PRKCD, PPP1CB, KCNMB2, GNAQ, MAPK3, ADRA1B, PLA2G6, GNAS, GUCY1B3, CALCRL, PLCB1, CACNA1D
Adherens junction	18	2.24E−05	ACTN4, LEF1, ACTN1, SMAD3, SNAI2, TCF7L2, SNAI1, IQGAP1, VCL, IGF1R, TJP1, EP300, RAC1, MAPK3, WASL, PTPN1, YES1, INSR
Oocyte meiosis	18	0.004111	ADCY1, MAP2K1, PPP2R5A, CPEB3, CAMK2G, BTRC, PPP1CB, IGF1R, CCNE1, MAD2L1, SLK, RPS6KA2, PPP2CA, MAPK3, YWHAQ, PPP3CA, CAMK2A, FBXW11
Amoebiasis	18	0.007719	COL4A3, IL1R1, ADCY1, TNF, ACTN4, ACTN1, ITGB2, COL5A2, IL10, VCL, GNAQ, LAMA5, IL12A, GNAS, SERPINB13, PLCB1, COL24A1, COL11A1
Platelet activation	18	0.022312	ADCY1, GNAI3, ROCK2, PTGS1, PRKG1, PPP1CB, COL5A2, GNAQ, MAPK12, FGA, MAPK3, GNAS, GUCY1B3, RAP1B, PLCB1, COL24A1, COL11A1, AKT3
Serotonergic synapse	18	0.023857	GNAI3, MAP2K1, PTGS1, MAOB, SLC6A4, HTR4, RAF1, KCNJ5, NRAS, APP, GNAQ, GNB2, GNG10, MAPK3, SLC18A2, GNAS, PLCB1, CACNA1D
Tight junction	18	0.037031	GNAI3, ACTN4, MPDZ, MRAS, PRKCH, ACTN1, AMOTL1, PRKCD, PTEN, CLDN15, NRAS, TJP1, PPP2CA, ASH1L, RAB13, YES1, AKT3, PPP2R2D
Estrogen signaling pathway	17	0.003032	ADCY1, GNAI3, MAP2K1, RAF1, PRKCD, KCNJ5, NRAS, FOS, GNAQ, SP1, JUN, MAPK3, CREB3L1, HBEGF, GNAS, PLCB1, AKT3
Glucagon signaling pathway	17	0.003732	CRTC2, CAMK2G, PHKA1, PRKAB2, PRKAB1, ACACA, PDE3B, FOXO1, SIRT1, EP300, GNAQ, CREB3L1, GNAS, PPP3CA, PLCB1, CAMK2A, AKT3
Choline metabolism in cancer	17	0.004129	MAP2K1, CHKB, RAF1, NRAS, FOS, HIF1A, SP1, DGKE, JUN, DGKG, MAPK3, RAC1, MAPK9, SLC5A7, WASL, PDGFD, AKT3
Insulin resistance	17	0.009491	CRTC2, TNF, PRKAB2, PRKAB1, FOXO1, PRKCD, PTEN, PPP1CB, PPP1R3D, RPS6KA2, MLX, CREB3L1, MAPK9, OGT, PTPN1, INSR, AKT3
Glutamatergic synapse	17	0.014278	SLC38A3, ADCY1, GNAI3, SLC38A2, GRIK5, GRIA4, HOMER1, SLC17A6, GNAQ, GRIA2, GNB2, GNG10, MAPK3, GNAS, PPP3CA, PLCB1, CACNA1D
Ubiquitin mediated proteolysis	17	0.077448	SYVN1, UBE3A, BTRC, UBE4B, CBL, BIRC6, UBE2H, HERC2, STUB1, CUL3, UBE2E3, CBLB, MAP3K1, WWP1, UBE2M, TRIM32, FBXW11
Prostate cancer	16	0.002635	E2F2, MAP2K1, FOXO1, RAF1, LEF1, PTEN, TCF7L2, NRAS, IGF1R, CCNE1, CDKN1A, EP300, MAPK3, CREB3L1, PDGFD, AKT3
Thyroid hormone signaling pathway	16	0.02557	KAT2B, MAP2K1, RAF1, FOXO1, MED13, ATP1A2, NRAS, NCOA1, NOTCH1, HIF1A, EP300, NCOA3, MAPK3, DIO1, PLCB1, AKT3
Inflammatory mediator regulation of TRP channels	16	0.058489	IL1R1, ADCY1, PTGER4, CAMK2G, TRPA1, PRKCH, PRKCD, PPP1CB, GNAQ, MAPK12, PLA2G6, MAPK9, GNAS, PLCB1, CAMK2A, MAP2K6
Osteoclast differentiation	16	0.058489	IL1R1, TNF, MAP2K1, CSF1, PPARG, FOS, TNFSF11, MAPK12, JUN, MAPK3, RAC1, MAPK9, PPP3CA, MAP2K6, AKT3, NFATC1
Long-term potentiation	15	3.92E−04	ADCY1, MAP2K1, CAMK2G, RAF1, PPP1CB, NRAS, EP300, GNAQ, GRIA2, RPS6KA2, MAPK3, RAP1B, PPP3CA, PLCB1, CAMK2A
Salmonella infection	15	0.0022	DYNC1I1, DYNC1LI2, ROCK2, ARPC1A, FOS, TJP1, MAPK12, ARPC2, JUN, MAPK3, RAC1, MAPK9, WASL, DYNC1H1, DYNC1I2
Gap junction	15	0.005529	ADCY1, GNAI3, MAP2K1, RAF1, LPAR1, PRKG1, NRAS, TJP1, GNAQ, MAP3K2, MAPK3, GNAS, GUCY1B3, PDGFD, PLCB1
Toll-like receptor signaling pathway	15	0.02171	TNF, MAP2K1, MAP2K4, CXCL9, FOS, MAPK12, JUN, RAC1, MAPK3, MAP3K8, IL12A, MAPK9, MAP2K6, AKT3, TRAF3
TNF signaling pathway	15	0.038777	TNF, DNM1L, MAP2K1, CSF1, MAP2K4, FOS, MAPK12, JUN, MAPK3, MAP3K8, CREB3L1, MAPK9, AKT3, MAP2K6, TRAF3
Cell cycle	15	0.093007	E2F2, E2F5, CDC14B, SMAD3, CDK7, CCNE1, CDKN1A, MAD2L1, EP300, RAD21, CCND2, YWHAQ, GADD45A, STAG2, TFDP1
Amphetamine addiction	14	0.001509	CAMK2G, MAOB, GRIA4, PPP1CB, SIRT1, FOS, GRIA2, JUN, SLC18A2, CREB3L1, GNAS, PPP3CA, CACNA1D, CAMK2A
Renal cell carcinoma	14	0.001509	MAP2K1, RAF1, HGF, PAK7, NRAS, CRKL, EP300, HIF1A, PAK4, JUN, RAC1, MAPK3, RAP1B, AKT3
Fc gamma R-mediated phagocytosis	14	0.011216	VAV3, MAP2K1, RAF1, ARF6, PRKCD, AMPH, ARPC1A, CRKL, ARPC2, MAPK3, RAC1, MARCKS, WASL, AKT3
GABAergic synapse	14	0.014861	GABRG1, SLC38A3, ADCY1, GABRA2, GNAI3, GABRA1, SLC38A2, SLC6A1, GNB2, TRAK2, GNG10, ABAT, CACNA1D, NSF
Long-term depression	13	0.002005	GNAI3, MAP2K1, RAF1, PRKG1, IGF1R, NRAS, GRIA2, GNAQ, PPP2CA, MAPK3, GNAS, GUCY1B3, PLCB1
Progesterone-mediated oocyte maturation	13	0.033215	ADCY1, GNAI3, MAP2K1, CPEB3, PDE3B, RAF1, IGF1R, MAD2L1, MAPK12, RPS6KA2, MAPK3, MAPK9, AKT3
Colorectal cancer	12	0.008821	FOS, MAP2K1, JUN, MAPK3, RAC1, MAPK9, RAF1, SMAD3, LEF1, TCF7L2, AKT3, AXIN1
Fc epsilon RI signaling pathway	12	0.013779	NRAS, TNF, VAV3, MAPK12, MAP2K1, MAPK3, MAP2K4, RAC1, MAPK9, RAF1, AKT3, MAP2K6
Melanoma	12	0.018702	NRAS, IGF1R, E2F2, CDKN1A, FGF7, MAP2K1, MAPK3, RAF1, PDGFD, HGF, PTEN, AKT3
Chronic myeloid leukemia	12	0.020602	NRAS, E2F2, CDKN1A, CBLB, CRKL, MAP2K1, MAPK3, CBL, RAF1, BCL2L1, RUNX1, AKT3
Prolactin signaling pathway	12	0.022641	NRAS, FOS, TNFSF11, MAPK12, MAP2K1, CCND2, MAPK3, MAPK9, RAF1, SOCS7, AKT3, CISH
Pertussis	12	0.024823	FOS, GNAI3, TNF, NOD1, MAPK12, JUN, MAPK3, IL12A, MAPK9, ITGB2, SERPING1, IL10
Insulin secretion	12	0.06389	ADCY1, GNAQ, CAMK2G, CREB3L1, GNAS, ATP1A2, VAMP2, PLCB1, CAMK2A, CACNA1D, ADCYAP1, KCNMB2
Circadian rhythm	11	6.68E−05	CRY2, NR1D1, BTRC, PRKAB2, PRKAB1, PER2, PER1, PER3, FBXW11, CLOCK, FBXL3
Glioma	11	0.025453	NRAS, IGF1R, E2F2, CDKN1A, MAP2K1, CAMK2G, MAPK3, RAF1, PTEN, CAMK2A, AKT3
B cell receptor signaling pathway	11	0.040119	NRAS, FOS, VAV3, MAP2K1, JUN, MAPK3, RAC1, RAF1, PPP3CA, AKT3, NFATC1
Salivary secretion	11	0.069082	CD38, ADCY1, GNAQ, SLC12A2, ADRA1B, GNAS, GUCY1B3, ATP1A2, VAMP2, PLCB1, PRKG1
Bacterial invasion of epithelial cells	11	0.074081	ARPC1A, CAV2, CBLB, CAV1, CRKL, ARPC2, CBL, RAC1, WASL, CLTC, VCL
Synaptic vesicle cycle	10	0.045841	ATP6V1C1, SYT1, CPLX2, SLC17A6, SLC18A2, ATP6V1B2, VAMP2, CLTC, UNC13C, NSF
Pancreatic cancer	10	0.059074	E2F2, MAP2K1, MAPK3, RAC1, MAPK9, RAF1, RALA, SMAD3, BCL2L1, AKT3
Arrhythmogenic right ventricular cardiomyopathy	10	0.092277	ACTN4, DMD, ITGA1, CACNB2, ACTN1, LEF1, CACNB4, ITGA4, CACNA1D, TCF7L2
Renin secretion	10	0.092277	GNAI3, GNAQ, PTGER4, PDE3B, GNAS, GUCY1B3, PPP3CA, PLCB1, CACNA1D, ADCYAP1
Vasopressin-regulated water reabsorption	9	0.015204	DYNC1I1, DYNC1LI2, CREB3L1, GNAS, VAMP2, DYNC1H1, DYNC1I2, NSF, AQP2
Endometrial cancer	9	0.04321	NRAS, MAP2K1, MAPK3, RAF1, LEF1, PTEN, TCF7L2, AKT3, AXIN1
Acute myeloid leukemia	9	0.062572	NRAS, MAP2K1, MAPK3, RUNX1T1, RAF1, LEF1, RUNX1, TCF7L2, AKT3
Malaria	8	0.071947	LRP1, TNF, COMP, IL12A, ITGB2, HGF, SDC2, IL10
Amyotrophic lateral sclerosis	8	0.093164	DERL1, TNF, GRIA2, MAPK12, RAC1, BCL2L1, PPP3CA, MAP2K6
Dorso-ventral axis formation	7	0.010038	NOTCH1, MAP2K1, CPEB3, ETS1, ETS2, MAPK3, ETV6
Nicotine addiction	7	0.082655	GABRG1, GABRA2, SLC17A6, GABRA1, GRIA2, CHRNA4, GRIA4
Bladder cancer	7	0.090875	NRAS, E2F2, CDKN1A, MAP2K1, MAPK3, RAF1, HBEGF
Thyroid cancer	6	0.067242	NRAS, MAP2K1, PPARG, MAPK3, LEF1, TCF7L2
Sulfur metabolism	4	0.046731	SQRDL, PAPSS1, BPNT1, PAPSS2

### Key modules analyses

**Figure 4:**
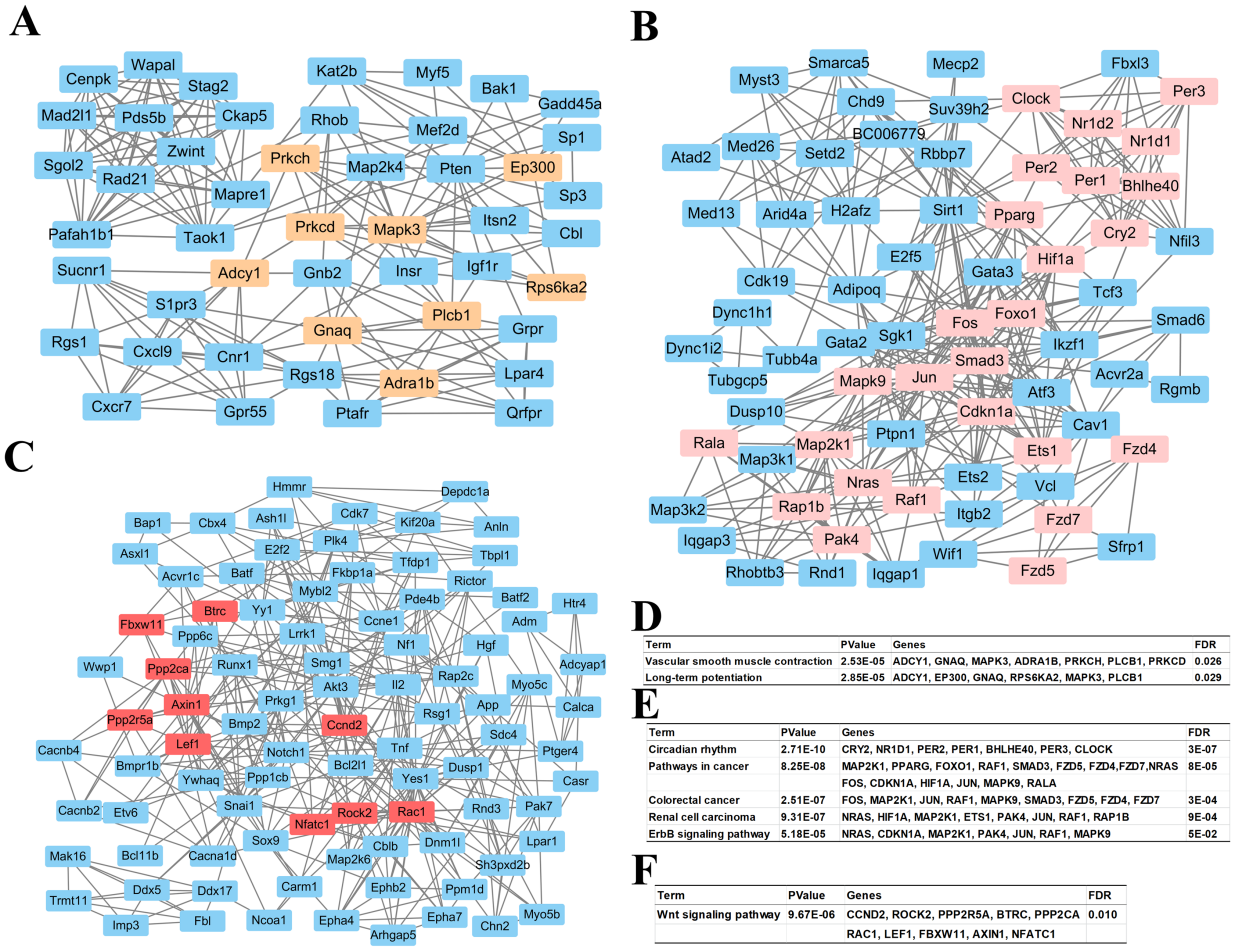
Top 3 modules of the protein–protein interaction network. (A) Module 1; (B) the enriched pathways of module 1; (C) module 2; (D) the enriched pathways of module 2; (E) module 3; (F) the enriched pathways of module 3. The rectangle in brown, pink and red represent the genes in high FDR signaling pathway in each module, respectively.

Based on information in the STRING database, all putative target genes constituted a PPI network that contained 1,360 nodes and 8,644 edges. The genes in the top three significant modules were further analyzed to evaluate their functions, and were involved in circadian rhythms, pathway in cancer, colorectal cancer, renal cell carcinoma, the ErbB signaling pathway, the wnt signaling pathway, vascular smooth muscle contraction, and long-term potentiation (Fig. 4).

### Validation of miRNA expression

**Figure 5:**
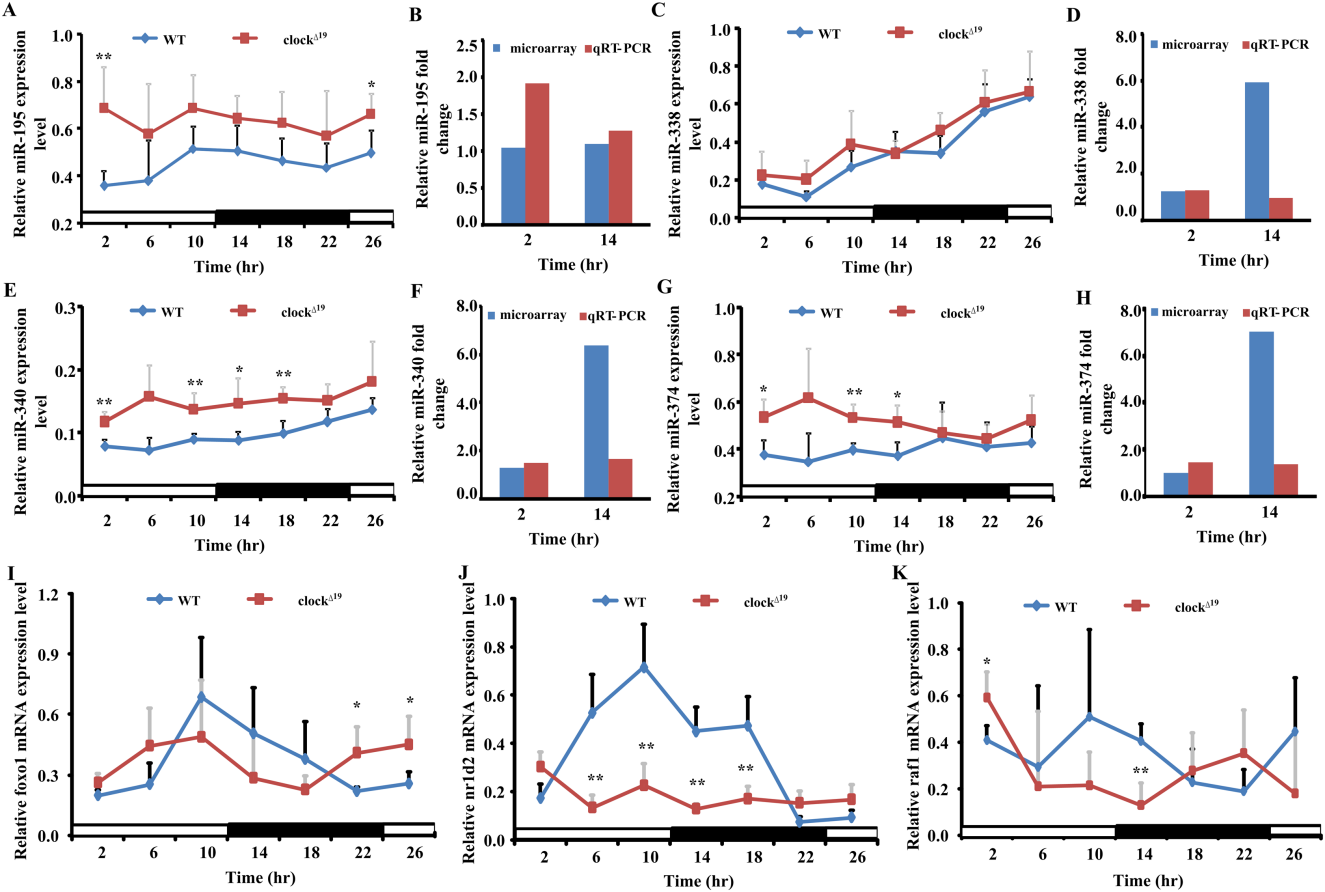
Four miRNAs and three mRNAs expression profiles in WT and *Clock* mutant mice liver. (A, C, E and G) show the expression of five differently expressed miRNAs in WT and clock mutant mice over 26 h (4 h intervals) in a 12 L/12 D photoperiod by qRT-PCR. Data points (means of 5 biological replicates ± SEM) were normalized using U6. (B, D, F and H) Showed the relative fold change of five miRNAs analyzed by microarray and qRT-PCR at two time points, respectively. In (B, D, F and H), the *y*-axis represents the relative fold change, the value greater than zero represents the upregulation and the value less than zero represents the down-regulation. The relative miRNAs fold change of microarray was analyzed by the fold change (FC) of *Clock* mutant and wild type (WT) mouse with mathematical formula log_2_^FC(*Clock*)/FC(WT)^. The relative miRNAs fold change of qPCR was analyzed by the value after normalization to U6 with the comparative threshold method. If the value of *Clock* mutant more than WT mouse, the upregulation fold change was calculated with the formula 2^−ΔΔCt(*Clock*)^/2^−ΔΔCt(WT)^. The down-regulation fold change was considered when the value of *Clock* mutant less than WT mouse and analyzed by the mathematical formula 2^−ΔΔCt (WT)^/2^−ΔΔCt (*Clock*)^. (I, J and K) Showed the expression level of three putative target genes in WT and *Clock* mutant mice over 26 h (4 h intervals) in a 12 L/12 D photoperiod by qRT-PCR, data points (means of 5 biological replicates ± SEM) were normalized using *gapdh*. *p < 0.05; **p< 0.01.

Five miRNAs (miR-195, miR-338, miR-340, miR-374 and miR-669d), whose putative target genes were involved in both pathways in cancer and circadian rhythms were validated by qRT-PCR to confirm the results of miRNAs microarray analyses. We also analyzed the expression levels of these miRNAs over a 26 h period at 4 h intervals to obtain a broader view of the rhythmic expression of these miRNAs. These miRNAs were all upregulated and consistent with the microarray results (Fig. 5). The expression levels of miR-195 and miR-340 retained a circadian manner in *Clock* mutant mice, despite the fact that their expression levels were greatly increased at all time points examined. The expression level of nr1d2 mRNA was down-regulated.

## Discussion

Circadian miRNAs involved in liver function represent a newly recognized level of the liver regulatory networks and the function of the biological clock. While many studies have demonstrated that CLOCK can regulate physiological processes in the liver by altering the expression of CCGs or physiological processes-related genes downstream of many cellular pathways, and microarray-based expression profiling of miRNAs in mouse liver at different time points has been used to identify circadian miRNAs, little is known about the molecular mechanism by which core clock genes coordinate with miRNAs to regulate liver function. In this study, we identified 104 miRNAs showing significant differential expression in the liver of *Clock* mutant mice (based on microarray analyses). We investigated their pathways and found that these miRNAs were mainly involved in cancer, PI3K-Akt signaling pathway, and the MAPK signaling pathway. Further investigation revealed that the hub genes were associated with circadian rhythms and pathway in cancer.

In our analyses, the liver-special miRNA, miR-122 (FC = 2.0009 at ZT2 and FC = 1.8112 at ZT14) was identified in liver tissue in absence of *Clock*, suggesting that our current strategy was appropriate for examining circadian miRNAs in the mouse liver although mature miRNAs did not show strong circadian oscillation. Furthermore, 14 miRNAs were altered at both analyzed time points, including miR-100, miR-195, miR-221, miR-338-5p, miR-34a, miR-340-3p, miR-374, miR-423-5p, miR-429, miR-455, miR-497, miR-532-5p, miR-669d and miR-802. Notably, except for miR-532-5p and miR-669d, the other miRNAs were previously identified to be involved in liver function. However, previous studies have only determined the association between these miRNAs and liver function, while the impact of circadian rhythms on miRNAs expression in the liver has not been examined. Thus, the identification of CLOCK-regulated miRNAs and their function in the circadian rhythm provides new insights.

Among the miRNA-mRNA pairs, four miRNAs directly targeted core circadian clock genes. This indicates that miRNAs may play a vital role in regulating circadian rhythms through core circadian clock genes. miR-34a targets *Per1* and is rhythmically expressed in cholangiocarcinoma cells and H69 cells, and its inhibition decreases proliferation, migration and invasion in cholangiocarcinoma cells (Han et al., 2016). Coupled with those results, our study suggests that the differentially expressed Clock-regulated miRNAs would construct a strong correlation network among miRNA expression, circadian rhythms, and liver function. Interestingly, although the expression levels of miR-100, miR-195, miR-221, miR-34a, miR-497 and miR-802 did not significantly differ at ZT2 and ZT14, they have been found to regulate hepatocellular carcinoma metastasis, hepatic insulin resistance, liver disease and other maladies. These results suggest that the miRNAs that do not show significant circadian oscillation are also worth of study.

Numerous studies have demonstrated that miRNAs might play important and extensive roles in liver development and regeneration in addition to contributing to or preventing chronic liver disease by directly or indirectly targeting cell cycle, proliferation or apoptosis genes. Zhou et al. (2014) found that miR-100 directly inhibits the expression of isoprenylcysteine carboxyl methyltransferase (ICMT) and ras-related C3 botulinum toxin substrate 1 (Rac1) by binding to their 3′ UTRs, and represses metastasis of hepatocellular carcinoma (HCC) cells. In another study, miR-100 suppressed the expression of mTOR and IGF-1R in HCC cells by binding to their 3′ UTR, and knockdown of mTOR or IGF-1R phenocopied the pro-autophagy effects of miR-100 (Ge et al., 2014). In our study, we also showed that Rac1 was regulated by miR-100. Although the suppression of IGF-1R by miR-100 was not observed, we found that Igf-1R was regulated by miR-340-3p. These differences may be due to the different prediction software used. IGF‑1R is a key component of the IGF axis that promotes cell proliferation, migration, and transformation. Increased expression of IGF-1R in HCC is closely associated with tumor progression (Scharf & Braulke, 2003). We predicted that miR-340-3p may inhibit proliferation in HCC cells by reducing IGF-1R levels. In a previous study, the target genes of miR-195 and miR-497 played important roles in increasing tumor life span, inhibiting tumor growth, and regulating cell cycle in HCC, and suppressing the growth of xenograft tumors in nude mice (Furuta et al., 2013). Consistent with these studies, the miR-195 targeting *Pcmt1* or *Cbx4* or *Btrc* or *Phf19* or *Ccne1*, and miR-497 targeting *Btrc* pairs were all observed in our results.

Gene co-expression network analysis is an essential tool for identifying the hub genes regulated by miRNAs. In our study, among the three modules screened using Cytoscape, the module pathways were mostly associated with the circadian rhythms. Studies of circadian rhythms provide important insights into the role of the circadian clock in liver physiology at the transcriptional level. However, recent proteomics studies have found that the levels of some metabolic enzymes cycle, although their transcript levels remain relatively constant throughout the day, indicating that post-transcriptional mechanisms might also be involved in circadian regulation of liver functions. Our results showed that miR-340-3p, miR-669d, miR-374 and miR-338-5p could directly target circadian genes (miR-340-3p targeting *Clock*, *Per1*, *Cry2*; miR-669d targeting *Per2*; miR-374 targeting *Per3*; miR-338-5p targeting *Nr1d1*), further supporting this idea. The other main pathway was pathway in cancer, and the genes in this pathway can impact tumor cell proliferation (PPARG, RAF1), regulate hepatic glucose and lipid metabolism (FOXO1), and decrease the viability and invasiveness of HCC cells (RALA) (Ezzeldin et al., 2014; O-Sullivan et al., 2015; Jeric et al., 2016; Li et al., 2017; Savic et al., 2016). This suggests that at the molecular level, CLOCK-regulated miRNAs may be involved in cancer initiation or progression by directly controlling cell proliferation, cell invasion, or metabolism-related genes in the mouse liver.

In summary, we identified changes in miRNA expression in *Clock* mutant mouse liver, and these CLOCK-regulated miRNAs had the ability to take part in liver function. Our results provide additional evidence to support the expanding research linking circadian rhythms dysfunction, miRNA expression, and liver function. Understanding the regulatory mechanism of *Clock* in the liver at the post-transcriptional level is critical importance for further highlighting the role of circadian miRNAs in peripheral organ or tissue. Our study focused only on 14 differentially expressed miRNAs, and analyses of the remaining miRNAs are necessary to fully understand the impact of circadian miRNAs on liver function and to identify biomarkers of liver disease.

## Conclusion

Our miRNA profile analyses identified a number of miRNAs that may be regulated by CLOCK and revealed these miRNAs linked to liver function. The functional roles of these miRNAs may affect the various physiological processes of the liver, and these data provide a reference for better understanding potential regulatory mechanisms in the liver.

## Supplemental Information

10.7717/peerj.8119/supp-1 Supplemental Information 1 The full list of differentially expressed miRNAs in liver of clock mutant at ZT2 with fold change (FC > 1). Clock mutant vs. WT.

10.7717/peerj.8119/supp-2 Supplemental Information 2 The full list of differentially expressed miRNAs in liver of clock mutant at ZT14 with fold change (FC > 1). Clock mutant vs. WT.

10.7717/peerj.8119/supp-3 Supplemental Information 3 The predicted target genes of the 14 differentially expressed miRNAs.

## Additional Information and Declarations

### Microarray Data Deposition

The following information was supplied regarding the deposition of microarray data: The microarray data is available in GEO: GSE140199.

### Data Availability

The following information was supplied regarding data availability: Raw data are provided in the Supplemental Materials.
