# Supplementary material for: Analysis of miRNA expression profiles in the liver of ClockΔ19 mutant mice
Source: PeerJ. 2019 Nov 28;7:e8119. doi: 10.7717/peerj.8119 (PMC6885354; doi:10.7717/peerj.8119)
Supplement: Supplemental Information 1 — Clock mutant vs. WT. [file peerj-07-8119-s001.docx]

Supplementary table 1.The full list of differentially expressed miRNAs in liver of clock mutant at ZT2 with fold change (FC >1).Clock mutant VS WT.

|  | MiRNA | FC |  | MiRNA | FC |  | MiRNA | FC |
| --- | --- | --- | --- | --- | --- | --- | --- | --- |
| 1 | mmu-miR-1 | -761.1791 | 66 | mmu-miR-223 | 1.3016 | 131 | mmu-miR-29c* | 1.7893 |
| 2 | mmu-miR-142-5p | -273.3303 | 67 | mmu-miR-212 | 1.3423 | 132 | mmu-miR-140* | 1.7978 |
| 3 | mmu-miR-133b | -230.5366 | 68 | mmu-miR-705 | 1.3514 | 133 | mmu-let-7f | 1.7998 |
| 4 | mmu-miR-669d | -159.8458 | 69 | mmu-miR-1196 | 1.3563 | 134 | mmu-miR-199a-5p | 1.8025 |
| 5 | mmu-miR-133a | -137.2458 | 70 | mmu-miR-1224 | 1.3765 | 135 | mmu-miR-28 | 1.803 |
| 6 | mmu-miR-429 | -135.4936 | 71 | mmu-miR-362-3p | 1.3795 | 136 | mmu-miR-148b | 1.8115 |
| 7 | mmu-miR-1894-3p | -128.1186 | 72 | mmu-miR-669a | 1.3891 | 137 | mmu-miR-361 | 1.817 |
| 8 | mmu-miR-197 | -120.8809 | 73 | mmu-miR-690 | 1.4003 | 138 | mmu-miR-697 | 1.8173 |
| 9 | mmu-miR-137 | -106.6092 | 74 | mmu-miR-805 | 1.4013 | 139 | mmu-miR-22 | 1.8179 |
| 10 | mmu-miR-210 | -78.5181 | 75 | mmu-miR-500 | 1.4307 | 140 | mmu-miR-199a-3p | 1.823 |
| 11 | mmu-miR-467a* | -75.692 | 76 | mmu-miR-702 | 1.431 | 141 | mmu-miR-335-5p | 1.8231 |
| 12 | mmu-miR-532-5p | -71.7111 | 77 | mmu-miR-466f-3p | 1.4312 | 142 | mmu-miR-22* | 1.8256 |
| 13 | mmu-miR-219 | -66.3245 | 78 | mmu-miR-21 | 1.4418 | 143 | mmu-miR-214 | 1.8265 |
| 14 | mmu-miR-669b | -64.6857 | 79 | mmu-miR-99b | 1.453 | 144 | mmu-miR-340-5p | 1.8554 |
| 15 | mmu-miR-141 | -63.7581 | 80 | mmu-miR-466c-5p | 1.4557 | 145 | mmu-miR-322 | 1.857 |
| 16 | mmu-miR-320 | -62.237 | 81 | mmu-miR-466g | 1.4593 | 146 | mmu-miR-20b | 1.8635 |
| 17 | mmu-miR-1894-5p | -61.3868 | 82 | mmu-let-7e | 1.4642 | 147 | mmu-miR-720 | 1.8649 |
| 18 | mmu-miR-487b | -57.7122 | 83 | mmu-miR-30c-2* | 1.4752 | 148 | mmu-miR-19b | 1.8671 |
| 19 | mmu-miR-455 | -57.3205 | 84 | mmu-miR-877* | 1.4776 | 149 | mmu-miR-29b | 1.8725 |
| 20 | mmu-miR-872 | -54.9073 | 85 | mmu-miR-466i | 1.4796 | 150 | mmu-miR-192 | 1.875 |
| 21 | mmu-miR-328 | -54.072 | 86 | mmu-miR-1895 | 1.4911 | 151 | mmu-miR-202-3p | 1.8798 |
| 22 | mmu-miR-291b-5p | -54.0331 | 87 | mmu-miR-17* | 1.4952 | 152 | mmu-miR-1904 | 1.8954 |
| 23 | mmu-miR-669e | -52.8361 | 88 | mmu-miR-712 | 1.5002 | 153 | mmu-let-7a | 1.9082 |
| 24 | mmu-miR-181a | -51.9831 | 89 | mmu-miR-101a | 1.5137 | 154 | mmu-miR-15a | 1.9104 |
| 25 | mmu-miR-671-5p | -42.8174 | 90 | mmu-miR-18a | 1.5146 | 155 | mmu-let-7d | 1.9109 |
| 26 | mmu-miR-466h | -40.1289 | 91 | mmu-miR-33 | 1.5208 | 156 | mmu-miR-24 | 1.9137 |
| 27 | mmu-miR-298 | -28.366 | 92 | mmu-miR-338-3p | 1.5305 | 157 | mmu-miR-126-3p | 1.9156 |
| 28 | mmu-miR-135a* | -28.0938 | 93 | mmu-miR-709 | 1.5305 | 158 | mmu-let-7g | 1.9202 |
| 29 | mmu-miR-188-5p | -27.3125 | 94 | mmu-miR-31 | 1.5309 | 159 | mmu-miR-467g | 1.9238 |
| 30 | mmu-miR-29b* | -27.2676 | 95 | mmu-miR-574-3p | 1.5394 | 160 | mmu-miR-1892 | 1.9325 |
| 31 | mmu-miR-125a-3p | -27.0697 | 96 | mmu-miR-467f | 1.5475 | 161 | mmu-miR-30d | 1.943 |
| 32 | mmu-miR-191* | -26.5133 | 97 | mmu-miR-27a | 1.5616 | 162 | mmu-miR-27b | 1.9484 |
| 33 | mmu-miR-346 | -25.8689 | 98 | mmu-miR-574-5p | 1.5623 | 163 | mmu-miR-194 | 1.9655 |
| 34 | mmu-miR-802 | -2.7938 | 99 | mmu-miR-1187 | 1.5696 | 164 | mmu-miR-20a | 1.9693 |
| 35 | mmu-let-7f* | -1.9705 | 100 | mmu-miR-345-5p | 1.5716 | 165 | mmu-miR-10a | 1.9786 |
| 36 | mmu-miR-1897-3p | -1.7432 | 101 | mmu-miR-98 | 1.5781 | 166 | mmu-miR-23b | 1.9906 |
| 37 | mmu-miR-1906 | -1.672 | 102 | mmu-miR-296-5p | 1.584 | 167 | mmu-miR-107 | 1.9979 |
| 38 | mmu-miR-1897-5p | -1.6147 | 103 | mmu-miR-148a | 1.5861 | 168 | mmu-miR-122 | 2.0009 |
| 39 | mmu-let-7b* | -1.4965 | 104 | mmu-miR-30a | 1.6182 | 169 | mmu-miR-101b | 2.0061 |
| 40 | mmu-miR-301a | -1.4309 | 105 | mmu-miR-203 | 1.6256 | 170 | mmu-miR-93 | 2.008 |
| 41 | mmu-miR-200b | -1.2954 | 106 | mmu-miR-19a | 1.6266 | 171 | mmu-miR-374 | 2.0122 |
|  | MiRNA | FC |  | MiRNA | FC |  | MiRNA | FC |
| 42 | mmu-miR-207 | -1.288 | 107 | mmu-miR-30e* | 1.6421 | 172 | mmu-miR-151-5p | 2.0132 |
| 43 | mmu-miR-483* | -1.2399 | 108 | mmu-miR-139-3p | 1.6424 | 173 | mmu-miR-185 | 2.0255 |
| 44 | mmu-miR-200a | -1.2296 | 109 | mmu-miR-669f | 1.645 | 174 | mmu-miR-423-5p | 2.0389 |
| 45 | mmu-miR-468 | -1.2188 | 110 | mmu-miR-30b | 1.6525 | 175 | mmu-miR-103 | 2.043 |
| 46 | mmu-miR-331-3p | -1.1714 | 111 | mmu-miR-378 | 1.6643 | 176 | mmu-miR-195 | 2.0635 |
| 47 | mmu-miR-31* | -1.0914 | 112 | mmu-miR-193 | 1.6724 | 177 | mmu-miR-16 | 2.0805 |
| 48 | mmu-miR-706 | -1.0743 | 113 | mmu-let-7i | 1.6741 | 178 | mmu-miR-497 | 2.0806 |
| 49 | mmu-miR-149 | -1.0285 | 114 | mmu-miR-669c | 1.6749 | 179 | mmu-let-7c | 2.1765 |
| 50 | mmu-miR-125a-5p | -1.0186 | 115 | mmu-miR-15b | 1.6788 | 180 | mmu-miR-25 | 2.1903 |
| 51 | mmu-miR-290-3p | -1.0064 | 116 | mmu-miR-30a* | 1.6928 | 181 | mmu-miR-92a | 2.2167 |
| 52 | mmu-miR-96 | 1.0198 | 117 | mmu-miR-106b | 1.7006 | 182 | mmu-miR-125b-5p | 2.2237 |
| 53 | mmu-miR-425 | 1.0255 | 118 | mmu-miR-23a | 1.7011 | 183 | mmu-miR-99a | 2.2451 |
| 54 | mmu-miR-378* | 1.0777 | 119 | mmu-miR-26b | 1.7191 | 184 | mmu-miR-144 | 2.2713 |
| 55 | mmu-miR-342-3p | 1.1101 | 120 | mmu-miR-29c | 1.7198 | 185 | mmu-miR-365 | 2.2813 |
| 56 | mmu-miR-146a | 1.1116 | 121 | mmu-miR-7a | 1.7307 | 186 | mmu-let-7b | 2.2895 |
| 57 | mmu-miR-142-3p | 1.1125 | 122 | mmu-miR-350 | 1.734 | 187 | mmu-miR-338-5p | 2.3313 |
| 58 | mmu-miR-199b* | 1.139 | 123 | mmu-miR-152 | 1.737 | 188 | mmu-miR-340-3p | 2.415 |
| 59 | mmu-miR-143 | 1.1608 | 124 | mmu-miR-26a | 1.7381 | 189 | mmu-miR-34a | 2.5785 |
| 60 | mmu-miR-689 | 1.1682 | 125 | mmu-miR-140 | 1.7399 | 190 | mmu-miR-221 | 2.6127 |
| 61 | mmu-miR-1198 | 1.2488 | 126 | mmu-miR-30e | 1.7414 | 191 | mmu-miR-100 | 2.6795 |
| 62 | mmu-miR-494 | 1.2559 | 127 | mmu-miR-29a | 1.745 | 192 | mmu-miR-139-5p | 2.7502 |
| 63 | mmu-miR-652 | 1.2661 | 128 | mmu-miR-126-5p | 1.7764 | 193 | mmu-miR-451 | 3.1432 |
| 64 | mmu-miR-669h-3p | 1.2837 | 129 | mmu-miR-30c | 1.7839 | 194 | mmu-miR-486 | 253.1871 |
| 65 | mmu-miR-130a | 1.2871 | 130 | mmu-miR-10b | 1.7882 |  |  |  |
