# Supplementary material for: Analysis of miRNA expression profiles in the liver of ClockΔ19 mutant mice
Source: PeerJ. 2019 Nov 28;7:e8119. doi: 10.7717/peerj.8119 (PMC6885354; doi:10.7717/peerj.8119)
Supplement: Supplemental Information 2 — Clock mutant vs. WT. [file peerj-07-8119-s002.docx]

Supplementary table 2.The full list of differentially expressed miRNAs in liver of clock mutant at ZT14 with fold change (FC >1).Clock mutant VS WT.

|  | **MiRNA** | **FC** |  | **MiRNA** | **FC** |  | **MiRNA** | **FC** |
| --- | --- | --- | --- | --- | --- | --- | --- | --- |
| 1 | mmu-miR-574-3p | -640.9941 | 54 | mmu-miR-30e | 1.4014 | 107 | mmu-miR-19b | 1.7527 |
| 2 | mmu-miR-1196 | -613.3541 | 55 | mmu-miR-362-3p | 1.4066 | 108 | mmu-miR-151-5p | 1.7620 |
| 3 | mmu-miR-669c | -608.4347 | 56 | mmu-miR-1892 | 1.4088 | 109 | mmu-miR-30a* | 1.7656 |
| 4 | mmu-miR-466g | -463.9785 | 57 | mmu-miR-101a | 1.4097 | 110 | mmu-miR-92a | 1.7662 |
| 5 | mmu-miR-669f | -347.7199 | 58 | mmu-miR-144 | 1.4190 | 111 | mmu-let-7c | 1.7789 |
| 6 | mmu-miR-466c-5p | -311.9278 | 59 | mmu-miR-106b | 1.4357 | 112 | mmu-miR-322 | 1.7813 |
| 7 | mmu-miR-669a | -253.3540 | 60 | mmu-miR-30a | 1.4526 | 113 | mmu-miR-25 | 1.7833 |
| 8 | mmu-miR-669d | -213.9050 | 61 | mmu-miR-30c | 1.4539 | 114 | mmu-miR-199b* | 1.7858 |
| 9 | mmu-miR-468 | -156.6158 | 62 | mmu-miR-30d | 1.4619 | 115 | mmu-miR-199a-5p | 1.7892 |
| 10 | mmu-miR-455 | -128.0480 | 63 | mmu-miR-1904 | 1.4701 | 116 | mmu-miR-122 | 1.8112 |
| 11 | mmu-miR-149 | -122.0470 | 64 | mmu-miR-22* | 1.4803 | 117 | mmu-miR-142-3p | 1.8151 |
| 12 | mmu-let-7f* | -109.8524 | 65 | mmu-miR-26b | 1.4871 | 118 | mmu-miR-143 | 1.8233 |
| 13 | mmu-miR-483* | -109.2050 | 66 | mmu-let-7i | 1.4876 | 119 | mmu-miR-10a | 1.8373 |
| 14 | mmu-miR-302b | -82.4306 | 67 | mmu-miR-148a | 1.4911 | 120 | mmu-miR-93 | 1.8974 |
| 15 | mmu-miR-207 | -53.3181 | 68 | mmu-miR-139-5p | 1.4993 | 121 | mmu-miR-361 | 1.9101 |
| 16 | mmu-miR-466f-3p | -7.4758 | 69 | mmu-miR-194 | 1.5024 | 122 | mmu-miR-99a | 1.9149 |
| 17 | mmu-miR-466i | -5.7110 | 70 | mmu-miR-148b | 1.5064 | 123 | mmu-miR-125b-5p | 1.9449 |
| 18 | mmu-miR-467f | -4.6940 | 71 | mmu-miR-15b | 1.5219 | 124 | mmu-miR-199a-3p | 1.9963 |
| 19 | mmu-miR-877* | -3.0182 | 72 | mmu-let-7d | 1.5441 | 125 | mmu-miR-20b | 2.0229 |
| 20 | mmu-miR-1187 | -2.6691 | 73 | mmu-miR-16 | 1.5526 | 126 | mmu-miR-497 | 2.0550 |
| 21 | mmu-miR-574-5p | -2.5782 | 74 | mmu-miR-27b | 1.5567 | 127 | mmu-miR-20a | 2.0634 |
| 22 | mmu-miR-802 | -2.1499 | 75 | mmu-let-7g | 1.5609 | 128 | mmu-miR-195 | 2.1270 |
| 23 | mmu-miR-706 | -2.0695 | 76 | mmu-miR-126-3p | 1.5611 | 129 | mmu-miR-494 | 2.1540 |
| 24 | mmu-miR-1897-3p | -2.0383 | 77 | mmu-miR-223 | 1.5615 | 130 | mmu-miR-140* | 2.1901 |
| 25 | mmu-let-7b* | -1.8251 | 78 | mmu-miR-31 | 1.5706 | 131 | mmu-miR-350 | 2.3735 |
| 26 | mmu-miR-689 | -1.8073 | 79 | mmu-miR-107 | 1.5731 | 132 | mmu-miR-200b | 2.4956 |
| 27 | mmu-miR-702 | -1.2885 | 80 | mmu-miR-103 | 1.5883 | 133 | mmu-miR-221 | 2.5242 |
| 28 | mmu-miR-1224 | -1.1418 | 81 | mmu-miR-21 | 1.5900 | 134 | mmu-miR-34a | 2.5247 |
| 29 | mmu-miR-378* | 1.1334 | 82 | mmu-miR-98 | 1.6011 | 135 | mmu-miR-30e* | 2.5738 |
| 30 | mmu-miR-720 | 1.1552 | 83 | mmu-miR-152 | 1.6047 | 136 | mmu-miR-100 | 2.6643 |
| 31 | mmu-miR-345-5p | 1.1710 | 84 | mmu-miR-340-5p | 1.6068 | 137 | mmu-miR-28 | 2.7031 |
| 32 | mmu-miR-805 | 1.2173 | 85 | mmu-miR-24 | 1.6081 | 138 | mmu-miR-200a | 2.9437 |
| 33 | mmu-miR-652 | 1.2331 | 86 | mmu-miR-101b | 1.6186 | 139 | mmu-miR-290-3p | 59.7184 |
| 34 | mmu-miR-500 | 1.2415 | 87 | mmu-miR-30c-2* | 1.6307 | 140 | mmu-miR-338-5p | 60.7003 |
| 35 | mmu-miR-1895 | 1.2569 | 88 | mmu-miR-29b | 1.6320 | 141 | mmu-miR-697 | 62.0710 |
| 36 | mmu-miR-378 | 1.2679 | 89 | mmu-miR-23a | 1.6380 | 142 | mmu-miR-340-3p | 83.1064 |
| 37 | mmu-miR-130a | 1.2729 | 90 | mmu-miR-23b | 1.6402 | 143 | mmu-miR-532-5p | 119.4200 |
| 38 | mmu-miR-203 | 1.2781 | 91 | mmu-let-7f | 1.6452 | 144 | mmu-miR-18a | 119.4950 |
| 39 | mmu-miR-709 | 1.2842 | 92 | mmu-miR-192 | 1.6513 | 145 | mmu-miR-296-5p | 125.7100 |
| 40 | mmu-miR-202-3p | 1.3090 | 93 | mmu-miR-27a | 1.6554 | 146 | mmu-miR-374 | 132.3890 |
| 41 | mmu-miR-212 | 1.3202 | 94 | mmu-miR-7a | 1.6585 | 147 | mmu-miR-29c* | 135.7150 |
|  | **MiRNA** | **FC** |  | **MiRNA** | **FC** |  | **MiRNA** | **FC** |
| 42 | mmu-miR-29c | 1.3363 | 95 | mmu-miR-19a | 1.6635 | 148 | mmu-miR-99b | 138.7420 |
| 43 | mmu-miR-425 | 1.3409 | 96 | mmu-miR-15a | 1.6660 | 149 | mmu-miR-301a | 148.0010 |
| 44 | mmu-miR-126-5p | 1.3410 | 97 | mmu-miR-193 | 1.6756 | 150 | mmu-miR-423-5p | 148.2080 |
| 45 | mmu-miR-185 | 1.3438 | 98 | mmu-let-7e | 1.6771 | 151 | mmu-miR-10b | 161.7690 |
| 46 | mmu-miR-331-3p | 1.3529 | 99 | mmu-let-7b | 1.6838 | 152 | mmu-miR-33 | 163.2010 |
| 47 | mmu-miR-22 | 1.3556 | 100 | mmu-miR-125a-5p | 1.6941 | 153 | mmu-miR-31* | 179.7160 |
| 48 | mmu-miR-690 | 1.3582 | 101 | mmu-miR-342-3p | 1.6989 | 154 | mmu-miR-214 | 181.8760 |
| 49 | mmu-miR-30b | 1.3757 | 102 | mmu-miR-365 | 1.7126 | 155 | mmu-miR-17* | 204.7430 |
| 50 | mmu-miR-451 | 1.3812 | 103 | mmu-let-7a | 1.7136 | 156 | mmu-miR-429 | 239.5100 |
| 51 | mmu-miR-26a | 1.3931 | 104 | mmu-miR-338-3p | 1.7202 | 157 | mmu-miR-96 | 358.8089 |
| 52 | mmu-miR-29a | 1.3963 | 105 | mmu-miR-140 | 1.7230 |  |  |  |
| 53 | mmu-miR-335-5p | 1.3992 | 106 | mmu-miR-146a | 1.7378 |  |  |  |
