# Supplementary material for: Analysis of miRNA expression profiles in the liver of ClockΔ19 mutant mice
Source: PeerJ. 2019 Nov 28;7:e8119. doi: 10.7717/peerj.8119 (PMC6885354; doi:10.7717/peerj.8119)
Supplement: Supplemental Information 3 [file peerj-07-8119-s003.docx]

Supplementary table 3. The predicted target genes of the 14 differentially expressed miRNAs.

| **Number** | **miRNA** | **Target gene** | **Number** | **miRNA** | **Target gene** |
| --- | --- | --- | --- | --- | --- |
| 1 | mmu-miR-34a | E2f5 | 892 | mmu-miR-374 | Pak1ip1 |
| 2 | mmu-miR-34a | Strn3 | 893 | mmu-miR-374 | Elovl2 |
| 3 | mmu-miR-34a | Dbc1 | 894 | mmu-miR-374 | Zfp91 |
| 4 | mmu-miR-34a | Lef1 | 895 | mmu-miR-374 | Plch1 |
| 5 | mmu-miR-34a | Zfhx4 | 896 | mmu-miR-374 | Pcnx |
| 6 | mmu-miR-34a | Pacs1 | 897 | mmu-miR-374 | Pde6a |
| 7 | mmu-miR-34a | Pkia | 898 | mmu-miR-374 | Ccnl1 |
| 8 | mmu-miR-34a | Fbxo30 | 899 | mmu-miR-374 | Dek |
| 9 | mmu-miR-34a | Vcl | 900 | mmu-miR-374 | Il12a |
| 10 | mmu-miR-34a | Dnm1l | 901 | mmu-miR-374 | Onecut2 |
| 11 | mmu-miR-34a | Mta2 | 902 | mmu-miR-374 | Mobkl3 |
| 12 | mmu-miR-34a | Marcks | 903 | mmu-miR-374 | Hes1 |
| 13 | mmu-miR-34a | Nrn1 | 904 | mmu-miR-374 | Bicd2 |
| 14 | mmu-miR-34a | Gmfb | 905 | mmu-miR-374 | Aldh1a1 |
| 15 | mmu-miR-34a | Numbl | 906 | mmu-miR-374 | Fut9 |
| 16 | mmu-miR-34a | Uhrf2 | 907 | mmu-miR-374 | Manea |
| 17 | mmu-miR-34a | Iqgap3 | 908 | mmu-miR-374 | Ccdc126 |
| 18 | mmu-miR-34a | Neto1 | 909 | mmu-miR-374 | Asb5 |
| 19 | mmu-miR-34a | Yy1 | 910 | mmu-miR-374 | Gria2 |
| 20 | mmu-miR-34a | Taf5 | 911 | mmu-miR-374 | Gpm6a |
| 21 | mmu-miR-34a | Map2k1 | 912 | mmu-miR-374 | Tdo2 |
| 22 | mmu-miR-34a | Notch1 | 913 | mmu-miR-374 | Nfil3 |
| 23 | mmu-miR-34a | Rarg | 914 | mmu-miR-374 | Ndufb3 |
| 24 | mmu-miR-34a | Nono | 915 | mmu-miR-374 | Pnrc1 |
| 25 | mmu-miR-34a | Erc1 | 916 | mmu-miR-374 | Eif3eip |
| 26 | mmu-miR-34a | Mras | 917 | mmu-miR-374 | Cbwd1 |
| 27 | mmu-miR-34a | Coro1c | 918 | mmu-miR-374 | Hoxa10 |
| 28 | mmu-miR-34a | Vamp2 | 919 | mmu-miR-374 | Hoxa11 |
| 29 | mmu-miR-34a | Figf | 920 | mmu-miR-374 | Mbd2 |
| 30 | mmu-miR-34a | Nrip3 | 921 | mmu-miR-374 | Faf2 |
| 31 | mmu-miR-34a | Ppp2r5a | 922 | mmu-miR-374 | Fhdc1 |
| 32 | mmu-miR-34a | Eif2s2 | 923 | mmu-miR-374 | Ocrl |
| 33 | mmu-miR-34a | Nup210 | 924 | mmu-miR-374 | Snx4 |
| 34 | mmu-miR-100 | Trib2 | 925 | mmu-miR-374 | Spock3 |
| 35 | mmu-miR-100 | Cdk7 | 926 | mmu-miR-374 | Gtpbp1 |
| 36 | mmu-miR-100 | Grhl1 | 927 | mmu-miR-374 | Znrf3 |
| 37 | mmu-miR-100 | Impdh1 | 928 | mmu-miR-374 | Psd3 |
| 38 | mmu-miR-100 | Mbnl1 | 929 | mmu-miR-374 | Rap2c |
| 39 | mmu-miR-100 | Hoxa1 | 930 | mmu-miR-374 | Ep300 |
| 40 | mmu-miR-100 | Vldlr | 931 | mmu-miR-374 | Pten |
| 41 | mmu-miR-100 | Comp | 932 | mmu-miR-374 | Fzd5 |
| 42 | mmu-miR-100 | Cubn | 933 | mmu-miR-374 | Gadd45a |
| 43 | mmu-miR-100 | Adcy1 | 934 | mmu-miR-374 | Mef2d |
| 44 | mmu-miR-100 | Smarca5 | 935 | mmu-miR-374 | Lrrtm3 |
| 45 | mmu-miR-100 | App | 936 | mmu-miR-374 | Lrrc58 |
| 46 | mmu-miR-100 | Sucla2 | 937 | mmu-miR-374 | Actn4 |
| 47 | mmu-miR-100 | Abhd2 | 938 | mmu-miR-374 | Slc39a12 |
| 48 | mmu-miR-100 | Ptpro | 939 | mmu-miR-374 | Fmr1 |
| 49 | mmu-miR-100 | 4922503N01Rik | 940 | mmu-miR-374 | Cacnb2 |
| 50 | mmu-miR-100 | Atpaf1 | 941 | mmu-miR-374 | Ctdsp1 |
| 51 | mmu-miR-100 | Jmjd3 | 942 | mmu-miR-374 | Bola3 |
| 52 | mmu-miR-100 | Pds5b | 943 | mmu-miR-374 | Gpr158 |
| 53 | mmu-miR-100 | Frap1 | 944 | mmu-miR-374 | Atp8b2 |
| 54 | mmu-miR-100 | Map2k6 | 945 | mmu-miR-374 | Msx1 |
| 55 | mmu-miR-100 | Cadm4 | 946 | mmu-miR-374 | Cdc42bpb |
| 56 | mmu-miR-100 | Ubap2 | 947 | mmu-miR-374 | Peli1 |
| 57 | mmu-miR-100 | Tgfbi | 948 | mmu-miR-374 | Spopl |
| 58 | mmu-miR-100 | Hcfc1 | 949 | mmu-miR-374 | Etfa |
| 59 | mmu-miR-100 | AC171108.2 | 950 | mmu-miR-374 | Crtc2 |
| 60 | mmu-miR-100 | Zswim5 | 951 | mmu-miR-374 | Wdr32 |
| 61 | mmu-miR-100 | Rac1 | 952 | mmu-miR-374 | Tmod1 |
| 62 | mmu-miR-195 | Tcfap2d | 953 | mmu-miR-374 | Pou4f2 |
| 63 | mmu-miR-195 | Plekhc1 | 954 | mmu-miR-374 | Epha4 |
| 64 | mmu-miR-195 | AC122398.2 | 955 | mmu-miR-374 | Itgb2 |
| 65 | mmu-miR-195 | Suz12 | 956 | mmu-miR-374 | Abce1 |
| 66 | mmu-miR-195 | Arhgap12 | 957 | mmu-miR-374 | Aak1 |
| 67 | mmu-miR-195 | Ywhaq | 958 | mmu-miR-374 | D12Ertd551e |
| 68 | mmu-miR-195 | Pcmt1 | 959 | mmu-miR-374 | Mecp2 |
| 69 | mmu-miR-195 | Dync1i1 | 960 | mmu-miR-374 | Ccdc88a |
| 70 | mmu-miR-195 | Capza2 | 961 | mmu-miR-374 | Elmod2 |
| 71 | mmu-miR-195 | Lrrn3 | 962 | mmu-miR-374 | Trappc10 |
| 72 | mmu-miR-195 | Arl2 | 963 | mmu-miR-374 | Cadm2 |
| 73 | mmu-miR-195 | Sil1 | 964 | mmu-miR-374 | Neo1 |
| 74 | mmu-miR-195 | Tbpl1 | 965 | mmu-miR-374 | Ankrd32 |
| 75 | mmu-miR-195 | Dmtf1 | 966 | mmu-miR-374 | Hr |
| 76 | mmu-miR-195 | Sema3a | 967 | mmu-miR-374 | Tmem161b |
| 77 | mmu-miR-195 | Hgf | 968 | mmu-miR-374 | Gabpa |
| 78 | mmu-miR-195 | Narg1 | 969 | mmu-miR-374 | Hapln1 |
| 79 | mmu-miR-195 | Srpr | 970 | mmu-miR-374 | Rbm47 |
| 80 | mmu-miR-195 | Sesn1 | 971 | mmu-miR-374 | Fundc2 |
| 81 | mmu-miR-195 | Runx1t1 | 972 | mmu-miR-374 | Kctd4 |
| 82 | mmu-miR-195 | Tmem55a | 973 | mmu-miR-374 | Brcc3 |
| 83 | mmu-miR-195 | Wwp1 | 974 | mmu-miR-374 | Lrig1 |
| 84 | mmu-miR-195 | Mobkl3 | 975 | mmu-miR-374 | Smad6 |
| 85 | mmu-miR-195 | Gpr63 | 976 | mmu-miR-374 | Homer1 |
| 86 | mmu-miR-195 | Hoxa10 | 977 | mmu-miR-374 | Lpar1 |
| 87 | mmu-miR-195 | Cdc37l1 | 978 | mmu-miR-374 | C630007B19Rik |
| 88 | mmu-miR-195 | Smad7 | 979 | mmu-miR-374 | Tnfsf11 |
| 89 | mmu-miR-195 | Casr | 980 | mmu-miR-374 | Gabrg1 |
| 90 | mmu-miR-195 | Serbp1 | 981 | mmu-miR-374 | Gng10 |
| 91 | mmu-miR-195 | Rabl3 | 982 | mmu-miR-374 | Dmd |
| 92 | mmu-miR-195 | C1ql3 | 983 | mmu-miR-374 | Eif4e3 |
| 93 | mmu-miR-195 | Setd3 | 984 | mmu-miR-374 | Elf1 |
| 94 | mmu-miR-195 | Arhgap20 | 985 | mmu-miR-374 | Neto2 |
| 95 | mmu-miR-195 | Bag5 | 986 | mmu-miR-374 | Gbx2 |
| 96 | mmu-miR-195 | Pou4f2 | 987 | mmu-miR-374 | Hmmr |
| 97 | mmu-miR-195 | Btrc | 988 | mmu-miR-374 | Tcf7l2 |
| 98 | mmu-miR-195 | Ccne1 | 989 | mmu-miR-374 | Lrrn1 |
| 99 | mmu-miR-195 | Rad23b | 990 | mmu-miR-374 | Pde4dip |
| 100 | mmu-miR-195 | Nup210 | 991 | mmu-miR-374 | Bhlhb2 |
| 101 | mmu-miR-195 | Lhx3 | 992 | mmu-miR-374 | Spin4 |
| 102 | mmu-miR-195 | Kctd8 | 993 | mmu-miR-374 | Setd5 |
| 103 | mmu-miR-195 | Ap3b1 | 994 | mmu-miR-374 | AI450540 |
| 104 | mmu-miR-195 | Shoc2 | 995 | mmu-miR-374 | Kcnj14 |
| 105 | mmu-miR-195 | Pcdh17 | 996 | mmu-miR-374 | Slc18a2 |
| 106 | mmu-miR-195 | Slitrk6 | 997 | mmu-miR-374 | Nhlh2 |
| 107 | mmu-miR-195 | Tarbp2 | 998 | mmu-miR-374 | Dlg3 |
| 108 | mmu-miR-195 | Raf1 | 999 | mmu-miR-374 | Sp1 |
| 109 | mmu-miR-195 | Ythdc1 | 1000 | mmu-miR-374 | St8sia4 |
| 110 | mmu-miR-195 | D4Bwg0951e | 1001 | mmu-miR-374 | Pappa |
| 111 | mmu-miR-195 | Ppap2a | 1002 | mmu-miR-374 | Trim32 |
| 112 | mmu-miR-195 | Prdm4 | 1003 | mmu-miR-374 | B230380D07Rik |
| 113 | mmu-miR-195 | Phf19 | 1004 | mmu-miR-374 | Dclre1b |
| 114 | mmu-miR-195 | Prkrir | 1005 | mmu-miR-374 | 2310035C23Rik |
| 115 | mmu-miR-195 | Ppp6c | 1006 | mmu-miR-374 | Rgs7bp |
| 116 | mmu-miR-195 | Cc2d1b | 1007 | mmu-miR-374 | Rfx7 |
| 117 | mmu-miR-195 | Wbp11 | 1008 | mmu-miR-374 | Zer1 |
| 118 | mmu-miR-195 | Acvr2a | 1009 | mmu-miR-374 | Galnact2 |
| 119 | mmu-miR-195 | Rnf10 | 1010 | mmu-miR-374 | Luzp2 |
| 120 | mmu-miR-195 | Usp33 | 1011 | mmu-miR-374 | Myo5c |
| 121 | mmu-miR-195 | Atxn2 | 1012 | mmu-miR-374 | A230046K03Rik |
| 122 | mmu-miR-195 | Akt3 | 1013 | mmu-miR-374 | Cyfip1 |
| 123 | mmu-miR-195 | Plxna2 | 1014 | mmu-miR-374 | Mpdz |
| 124 | mmu-miR-195 | Slc6a4 | 1015 | mmu-miR-374 | Tmod3 |
| 125 | mmu-miR-195 | Wsb1 | 1016 | mmu-miR-374 | Herc2 |
| 126 | mmu-miR-195 | Usp42 | 1017 | mmu-miR-374 | Sec24a |
| 127 | mmu-miR-195 | Ap1gbp1 | 1018 | mmu-miR-374 | En1 |
| 128 | mmu-miR-195 | Ppm1d | 1019 | mmu-miR-374 | Tmem77 |
| 129 | mmu-miR-195 | Cox11 | 1020 | mmu-miR-374 | Skiv2l2 |
| 130 | mmu-miR-195 | Dll4 | 1021 | mmu-miR-374 | Ube3a |
| 131 | mmu-miR-195 | Ube4b | 1022 | mmu-miR-374 | Ereg |
| 132 | mmu-miR-195 | Nfe2l1 | 1023 | mmu-miR-374 | Hspa4 |
| 133 | mmu-miR-195 | Cops2 | 1024 | mmu-miR-374 | Zcchc10 |
| 134 | mmu-miR-195 | Pak7 | 1025 | mmu-miR-374 | Aff4 |
| 135 | mmu-miR-195 | Tasp1 | 1026 | mmu-miR-374 | Parp8 |
| 136 | mmu-miR-195 | Helz | 1027 | mmu-miR-374 | Tjp1 |
| 137 | mmu-miR-195 | Cbx4 | 1028 | mmu-miR-374 | Cbfb |
| 138 | mmu-miR-195 | Iars | 1029 | mmu-miR-374 | Tbx22 |
| 139 | mmu-miR-195 | Cops7b | 1030 | mmu-miR-374 | Hdx |
| 140 | mmu-miR-195 | C1qtnf2 | 1031 | mmu-miR-374 | Dmrta1 |
| 141 | mmu-miR-195 | Col24a1 | 1032 | mmu-miR-374 | Il10 |
| 142 | mmu-miR-221 | Rab1 | 1033 | mmu-miR-374 | Acsl6 |
| 143 | mmu-miR-221 | Shprh | 1034 | mmu-miR-374 | Chm |
| 144 | mmu-miR-221 | Fbxo30 | 1035 | mmu-miR-374 | Nr1h4 |
| 145 | mmu-miR-221 | 5730410E15Rik | 1036 | mmu-miR-374 | Fnip1 |
| 146 | mmu-miR-221 | Capn7 | 1037 | mmu-miR-374 | Cdc42se2 |
| 147 | mmu-miR-221 | Tox | 1038 | mmu-miR-374 | Gk2 |
| 148 | mmu-miR-221 | Ptx3 | 1039 | mmu-miR-374 | Hel308 |
| 149 | mmu-miR-221 | Phf2 | 1040 | mmu-miR-374 | Col11a1 |
| 150 | mmu-miR-221 | Pdcd10 | 1041 | mmu-miR-374 | Aff1 |
| 151 | mmu-miR-221 | Fos | 1042 | mmu-miR-374 | Dusp6 |
| 152 | mmu-miR-221 | Wdr40a | 1043 | mmu-miR-374 | 1700129I04Rik |
| 153 | mmu-miR-221 | Gabra2 | 1044 | mmu-miR-374 | Tat |
| 154 | mmu-miR-221 | Irx5 | 1045 | mmu-miR-374 | Tmtc2 |
| 155 | mmu-miR-221 | Stap1 | 1046 | mmu-miR-374 | Ntf3 |
| 156 | mmu-miR-221 | Chsy1 | 1047 | mmu-miR-374 | Pde4b |
| 157 | mmu-miR-221 | Ap3b2 | 1048 | mmu-miR-374 | Mcart6 |
| 158 | mmu-miR-221 | 2610101N10Rik | 1049 | mmu-miR-374 | Syt1 |
| 159 | mmu-miR-221 | Sacm1l | 1050 | mmu-miR-374 | Rab38 |
| 160 | mmu-miR-221 | Depdc7 | 1051 | mmu-miR-374 | Rnf128 |
| 161 | mmu-miR-338-5p | Cpeb3 | 1052 | mmu-miR-374 | Oma1 |
| 162 | mmu-miR-338-5p | Map3k8 | 1053 | mmu-miR-374 | Kcnc2 |
| 163 | mmu-miR-338-5p | Papd1 | 1054 | mmu-miR-374 | Ppap2b |
| 164 | mmu-miR-338-5p | 0610009D07Rik | 1055 | mmu-miR-374 | Trhde |
| 165 | mmu-miR-338-5p | Ptger4 | 1056 | mmu-miR-374 | Ltbp1 |
| 166 | mmu-miR-338-5p | Mkx | 1057 | mmu-miR-374 | Tmed5 |
| 167 | mmu-miR-338-5p | Saps3 | 1058 | mmu-miR-374 | Dr1 |
| 168 | mmu-miR-338-5p | Gdi2 | 1059 | mmu-miR-374 | Pitx2 |
| 169 | mmu-miR-338-5p | Mycn | 1060 | mmu-miR-374 | Qpct |
| 170 | mmu-miR-338-5p | Rock2 | 1061 | mmu-miR-374 | Rsf1 |
| 171 | mmu-miR-338-5p | Mtmr12 | 1062 | mmu-miR-374 | Rgs18 |
| 172 | mmu-miR-338-5p | Ywhaq | 1063 | mmu-miR-374 | B830045N13Rik |
| 173 | mmu-miR-338-5p | Klf11 | 1064 | mmu-miR-374 | Rc3h2 |
| 174 | mmu-miR-338-5p | Slc4a7 | 1065 | mmu-miR-374 | Tmem108 |
| 175 | mmu-miR-338-5p | Myct1 | 1066 | mmu-miR-374 | Chrdl1 |
| 176 | mmu-miR-338-5p | Rnf138 | 1067 | mmu-miR-374 | Sfrs7 |
| 177 | mmu-miR-338-5p | Syne1 | 1068 | mmu-miR-374 | Alg13 |
| 178 | mmu-miR-338-5p | Bet1 | 1069 | mmu-miR-374 | Rap1b |
| 179 | mmu-miR-338-5p | Lig4 | 1070 | mmu-miR-374 | Etv6 |
| 180 | mmu-miR-338-5p | Ust | 1071 | mmu-miR-374 | Pik3r4 |
| 181 | mmu-miR-338-5p | Sash1 | 1072 | mmu-miR-374 | Pgm2l1 |
| 182 | mmu-miR-338-5p | Amotl1 | 1073 | mmu-miR-374 | Prepl |
| 183 | mmu-miR-338-5p | Emp2 | 1074 | mmu-miR-374 | Npl |
| 184 | mmu-miR-338-5p | Ythdf3 | 1075 | mmu-miR-374 | Iscu |
| 185 | mmu-miR-338-5p | Hdac9 | 1076 | mmu-miR-374 | Adcyap1 |
| 186 | mmu-miR-338-5p | Epdr1 | 1077 | mmu-miR-374 | Iqsec2 |
| 187 | mmu-miR-338-5p | Mtfr1 | 1078 | mmu-miR-374 | Slc38a3 |
| 188 | mmu-miR-338-5p | Ahr | 1079 | mmu-miR-374 | Galnt13 |
| 189 | mmu-miR-338-5p | Cav2 | 1080 | mmu-miR-374 | Ermn |
| 190 | mmu-miR-338-5p | Azin1 | 1081 | mmu-miR-374 | Map2k4 |
| 191 | mmu-miR-338-5p | Gpr126 | 1082 | mmu-miR-374 | Ctbs |
| 192 | mmu-miR-338-5p | Atp6v1c1 | 1083 | mmu-miR-374 | Stk38l |
| 193 | mmu-miR-338-5p | Rgmb | 1084 | mmu-miR-374 | Prrx1 |
| 194 | mmu-miR-338-5p | Capza2 | 1085 | mmu-miR-374 | Reps2 |
| 195 | mmu-miR-338-5p | Syvn1 | 1086 | mmu-miR-374 | Vamp2 |
| 196 | mmu-miR-338-5p | Angpt1 | 1087 | mmu-miR-374 | Lrriq3 |
| 197 | mmu-miR-338-5p | Il22ra2 | 1088 | mmu-miR-374 | Ttc21b |
| 198 | mmu-miR-338-5p | Slc35d3 | 1089 | mmu-miR-374 | Setd2 |
| 199 | mmu-miR-338-5p | Pex7 | 1090 | mmu-miR-374 | Frmpd4 |
| 200 | mmu-miR-338-5p | Paip2 | 1091 | mmu-miR-374 | Eif4g2 |
| 201 | mmu-miR-338-5p | Snai2 | 1092 | mmu-miR-374 | Pde3b |
| 202 | mmu-miR-338-5p | Arf4 | 1093 | mmu-miR-374 | Dusp10 |
| 203 | mmu-miR-338-5p | Efcab1 | 1094 | mmu-miR-374 | Ube2e3 |
| 204 | mmu-miR-338-5p | Arhgap5 | 1095 | mmu-miR-374 | Itga4 |
| 205 | mmu-miR-338-5p | Csmd1 | 1096 | mmu-miR-374 | Neurod1 |
| 206 | mmu-miR-338-5p | Il17rd | 1097 | mmu-miR-374 | Bpnt1 |
| 207 | mmu-miR-338-5p | Wasl | 1098 | mmu-miR-374 | Bsdc1 |
| 208 | mmu-miR-338-5p | Gcc1 | 1099 | mmu-miR-374 | Zdhhc5 |
| 209 | mmu-miR-338-5p | Kcnmb2 | 1100 | mmu-miR-374 | Plxna2 |
| 210 | mmu-miR-338-5p | Lrrc4 | 1101 | mmu-miR-374 | Brwd2 |
| 211 | mmu-miR-338-5p | Derl1 | 1102 | mmu-miR-374 | Ahdc1 |
| 212 | mmu-miR-338-5p | Atad2 | 1103 | mmu-miR-374 | Gnb2 |
| 213 | mmu-miR-338-5p | Pnn | 1104 | mmu-miR-374 | Mafk |
| 214 | mmu-miR-338-5p | Ctgf | 1105 | mmu-miR-374 | Dusp8 |
| 215 | mmu-miR-338-5p | Ahcyl2 | 1106 | mmu-miR-374 | Ece1 |
| 216 | mmu-miR-338-5p | Abca3 | 1107 | mmu-miR-374 | Nptx2 |
| 217 | mmu-miR-338-5p | Steap4 | 1108 | mmu-miR-374 | Ppm1d |
| 218 | mmu-miR-338-5p | Spry4 | 1109 | mmu-miR-374 | Med13 |
| 219 | mmu-miR-338-5p | Spg3a | 1110 | mmu-miR-374 | Fbxo42 |
| 220 | mmu-miR-338-5p | Nxf1 | 1111 | mmu-miR-374 | Spred1 |
| 221 | mmu-miR-338-5p | Hnrnpul2 | 1112 | mmu-miR-374 | Hlf |
| 222 | mmu-miR-338-5p | Xk | 1113 | mmu-miR-374 | Tom1l1 |
| 223 | mmu-miR-338-5p | Trmt11 | 1114 | mmu-miR-374 | Ube4b |
| 224 | mmu-miR-338-5p | Dynlt3 | 1115 | mmu-miR-374 | Per3 |
| 225 | mmu-miR-338-5p | Ncoa7 | 1116 | mmu-miR-374 | Eif3j |
| 226 | mmu-miR-338-5p | Txndc1 | 1117 | mmu-miR-374 | Rer1 |
| 227 | mmu-miR-338-5p | Sema3e | 1118 | mmu-miR-374 | Cops2 |
| 228 | mmu-miR-338-5p | Tspan7 | 1119 | mmu-miR-374 | Fgf7 |
| 229 | mmu-miR-338-5p | Cald1 | 1120 | mmu-miR-374 | Ap4e1 |
| 230 | mmu-miR-338-5p | BB146404 | 1121 | mmu-miR-374 | Mlx |
| 231 | mmu-miR-338-5p | Mtpn | 1122 | mmu-miR-374 | Txndc13 |
| 232 | mmu-miR-338-5p | Lamp3 | 1123 | mmu-miR-374 | Nsf |
| 233 | mmu-miR-338-5p | Sema6a | 1124 | mmu-miR-374 | Fkbp1a |
| 234 | mmu-miR-338-5p | Hif1a | 1125 | mmu-miR-374 | Slc16a6 |
| 235 | mmu-miR-338-5p | Ptpn12 | 1126 | mmu-miR-374 | Abca8b |
| 236 | mmu-miR-338-5p | Bmpr1a | 1127 | mmu-miR-374 | Eif2s2 |
| 237 | mmu-miR-338-5p | Fgl2 | 1128 | mmu-miR-374 | Map2k6 |
| 238 | mmu-miR-338-5p | Luc7l2 | 1129 | mmu-miR-374 | Stk4 |
| 239 | mmu-miR-338-5p | Ets1 | 1130 | mmu-miR-374 | Adnp |
| 240 | mmu-miR-338-5p | Rbm13 | 1131 | mmu-miR-374 | Tmepai |
| 241 | mmu-miR-338-5p | Fundc1 | 1132 | mmu-miR-374 | Metrnl |
| 242 | mmu-miR-338-5p | Nck2 | 1133 | mmu-miR-374 | Lama5 |
| 243 | mmu-miR-338-5p | Agk | 1134 | mmu-miR-374 | Ythdf1 |
| 244 | mmu-miR-338-5p | Isoc1 | 1135 | mmu-miR-374 | BC006779 |
| 245 | mmu-miR-338-5p | Axin1 | 1136 | mmu-miR-374 | Nin |
| 246 | mmu-miR-338-5p | Zfp182 | 1137 | mmu-miR-374 | Nfkbiz |
| 247 | mmu-miR-338-5p | Qrsl1 | 1138 | mmu-miR-374 | Eif3a |
| 248 | mmu-miR-338-5p | Sucnr1 | 1139 | mmu-miR-374 | Tbc1d8b |
| 249 | mmu-miR-338-5p | Sfrs10 | 1140 | mmu-miR-423-5p | Rai14 |
| 250 | mmu-miR-338-5p | Mbnl1 | 1141 | mmu-miR-423-5p | Golph3 |
| 251 | mmu-miR-338-5p | Dusp1 | 1142 | mmu-miR-423-5p | Ppp1r9a |
| 252 | mmu-miR-338-5p | Dgkg | 1143 | mmu-miR-423-5p | Pkia |
| 253 | mmu-miR-338-5p | Slc7a2 | 1144 | mmu-miR-423-5p | Sdc2 |
| 254 | mmu-miR-338-5p | Obfc2a | 1145 | mmu-miR-423-5p | Sash1 |
| 255 | mmu-miR-338-5p | Slc26a2 | 1146 | mmu-miR-423-5p | Hbp1 |
| 256 | mmu-miR-338-5p | Adipoq | 1147 | mmu-miR-423-5p | Clec16a |
| 257 | mmu-miR-338-5p | Ptx3 | 1148 | mmu-miR-423-5p | Grhl2 |
| 258 | mmu-miR-338-5p | Mstn | 1149 | mmu-miR-423-5p | Tfdp1 |
| 259 | mmu-miR-338-5p | Gnaq | 1150 | mmu-miR-423-5p | Yars2 |
| 260 | mmu-miR-338-5p | Ormdl1 | 1151 | mmu-miR-423-5p | Hfe |
| 261 | mmu-miR-338-5p | Zfp647 | 1152 | mmu-miR-423-5p | Opn1sw |
| 262 | mmu-miR-338-5p | Snrpc | 1153 | mmu-miR-423-5p | Clcn4-2 |
| 263 | mmu-miR-338-5p | Il13ra1 | 1154 | mmu-miR-423-5p | Hic2 |
| 264 | mmu-miR-338-5p | Id4 | 1155 | mmu-miR-423-5p | Rell2 |
| 265 | mmu-miR-338-5p | Il12a | 1156 | mmu-miR-423-5p | 2410127E18Rik |
| 266 | mmu-miR-338-5p | Snx25 | 1157 | mmu-miR-423-5p | Gnpda1 |
| 267 | mmu-miR-338-5p | Gm1040 | 1158 | mmu-miR-423-5p | Bap1 |
| 268 | mmu-miR-338-5p | Serinc1 | 1159 | mmu-miR-423-5p | Tspan7 |
| 269 | mmu-miR-338-5p | Mars2 | 1160 | mmu-miR-423-5p | Lonrf2 |
| 270 | mmu-miR-338-5p | Satb2 | 1161 | mmu-miR-423-5p | Abcc5 |
| 271 | mmu-miR-338-5p | Bicd2 | 1162 | mmu-miR-423-5p | Il1r1 |
| 272 | mmu-miR-338-5p | Tmem44 | 1163 | mmu-miR-423-5p | Zbtb24 |
| 273 | mmu-miR-338-5p | Zfand5 | 1164 | mmu-miR-423-5p | Nrn1 |
| 274 | mmu-miR-338-5p | 5730446C15Rik | 1165 | mmu-miR-423-5p | Rab40c |
| 275 | mmu-miR-338-5p | Tmem2 | 1166 | mmu-miR-423-5p | Nrgn |
| 276 | mmu-miR-338-5p | Pmaip1 | 1167 | mmu-miR-423-5p | Mme |
| 277 | mmu-miR-338-5p | Epha7 | 1168 | mmu-miR-423-5p | Nudt3 |
| 278 | mmu-miR-338-5p | Ccdc126 | 1169 | mmu-miR-423-5p | Aof1 |
| 279 | mmu-miR-338-5p | Vegfc | 1170 | mmu-miR-423-5p | S1pr3 |
| 280 | mmu-miR-338-5p | Gria2 | 1171 | mmu-miR-423-5p | Cdkn1a |
| 281 | mmu-miR-338-5p | Hpgd | 1172 | mmu-miR-423-5p | Thy1 |
| 282 | mmu-miR-338-5p | Fbxo8 | 1173 | mmu-miR-423-5p | Ccdc97 |
| 283 | mmu-miR-338-5p | Tctex1d2 | 1174 | mmu-miR-423-5p | Faf2 |
| 284 | mmu-miR-338-5p | Msx2 | 1175 | mmu-miR-423-5p | Pla2g6 |
| 285 | mmu-miR-338-5p | Cbl | 1176 | mmu-miR-423-5p | Ocrl |
| 286 | mmu-miR-338-5p | Cnr1 | 1177 | mmu-miR-423-5p | Hdgf |
| 287 | mmu-miR-338-5p | Stag2 | 1178 | mmu-miR-423-5p | Samd4b |
| 288 | mmu-miR-338-5p | Sh2d1a | 1179 | mmu-miR-423-5p | Tmem150 |
| 289 | mmu-miR-338-5p | Hoxa10 | 1180 | mmu-miR-423-5p | Atp11c |
| 290 | mmu-miR-338-5p | Ap1b1 | 1181 | mmu-miR-423-5p | Rcbtb1 |
| 291 | mmu-miR-338-5p | Smarca2 | 1182 | mmu-miR-423-5p | Gramd4 |
| 292 | mmu-miR-338-5p | Chn2 | 1183 | mmu-miR-423-5p | Ttll4 |
| 293 | mmu-miR-338-5p | Fzd7 | 1184 | mmu-miR-423-5p | Lmf2 |
| 294 | mmu-miR-338-5p | Galc | 1185 | mmu-miR-423-5p | Tspan3 |
| 295 | mmu-miR-338-5p | Suv39h2 | 1186 | mmu-miR-423-5p | Hmg20a |
| 296 | mmu-miR-338-5p | Ctla4 | 1187 | mmu-miR-423-5p | Ankrd27 |
| 297 | mmu-miR-338-5p | Hdhd2 | 1188 | mmu-miR-423-5p | Tshz3 |
| 298 | mmu-miR-338-5p | Prkg1 | 1189 | mmu-miR-423-5p | Cops7b |
| 299 | mmu-miR-338-5p | Mbnl3 | 1190 | mmu-miR-423-5p | Sh3pxd2b |
| 300 | mmu-miR-338-5p | Ep300 | 1191 | mmu-miR-423-5p | Slc25a30 |
| 301 | mmu-miR-338-5p | Yes1 | 1192 | mmu-miR-423-5p | Atp5d |
| 302 | mmu-miR-338-5p | Eid2 | 1193 | mmu-miR-423-5p | Gfra1 |
| 303 | mmu-miR-338-5p | Hprt1 | 1194 | mmu-miR-423-5p | Gjb1 |
| 304 | mmu-miR-338-5p | 4632404H22Rik | 1195 | mmu-miR-423-5p | Celsr2 |
| 305 | mmu-miR-338-5p | Gata3 | 1196 | mmu-miR-423-5p | Tubb4 |
| 306 | mmu-miR-338-5p | Smad5 | 1197 | mmu-miR-423-5p | Furin |
| 307 | mmu-miR-338-5p | Sirt1 | 1198 | mmu-miR-423-5p | Sf3b3 |
| 308 | mmu-miR-338-5p | Fbxo27 | 1199 | mmu-miR-423-5p | Mras |
| 309 | mmu-miR-338-5p | Tshz1 | 1200 | mmu-miR-423-5p | Birc6 |
| 310 | mmu-miR-338-5p | Dicer1 | 1201 | mmu-miR-423-5p | Ppap2b |
| 311 | mmu-miR-338-5p | Cyp26a1 | 1202 | mmu-miR-423-5p | Ank2 |
| 312 | mmu-miR-338-5p | Atp11c | 1203 | mmu-miR-423-5p | Gns |
| 313 | mmu-miR-338-5p | Mreg | 1204 | mmu-miR-423-5p | Iqsec2 |
| 314 | mmu-miR-338-5p | Dlat | 1205 | mmu-miR-423-5p | 6430517E21Rik |
| 315 | mmu-miR-338-5p | Slc39a12 | 1206 | mmu-miR-423-5p | Sema3f |
| 316 | mmu-miR-338-5p | Nat13 | 1207 | mmu-miR-423-5p | Trim68 |
| 317 | mmu-miR-338-5p | Nsun6 | 1208 | mmu-miR-423-5p | Mobkl2c |
| 318 | mmu-miR-338-5p | Ikzf1 | 1209 | mmu-miR-423-5p | Baz2a |
| 319 | mmu-miR-338-5p | Bcl11b | 1210 | mmu-miR-423-5p | Sft2d2 |
| 320 | mmu-miR-338-5p | Boc | 1211 | mmu-miR-423-5p | Ankrd52 |
| 321 | mmu-miR-338-5p | Gtpbp8 | 1212 | mmu-miR-423-5p | Rragc |
| 322 | mmu-miR-338-5p | Mllt10 | 1213 | mmu-miR-423-5p | Gorasp1 |
| 323 | mmu-miR-338-5p | Atxn10 | 1214 | mmu-miR-423-5p | Smtnl2 |
| 324 | mmu-miR-338-5p | Bmi1 | 1215 | mmu-miR-423-5p | Sbk1 |
| 325 | mmu-miR-338-5p | Hmgb3 | 1216 | mmu-miR-423-5p | Dlgap3 |
| 326 | mmu-miR-338-5p | Npat | 1217 | mmu-miR-423-5p | Atf3 |
| 327 | mmu-miR-338-5p | C1d | 1218 | mmu-miR-423-5p | Clip2 |
| 328 | mmu-miR-338-5p | Meis1 | 1219 | mmu-miR-423-5p | Eln |
| 329 | mmu-miR-338-5p | Rab1 | 1220 | mmu-miR-423-5p | Laptm5 |
| 330 | mmu-miR-338-5p | Traf3 | 1221 | mmu-miR-423-5p | Upk3b |
| 331 | mmu-miR-338-5p | Klf2 | 1222 | mmu-miR-423-5p | Tsc22d4 |
| 332 | mmu-miR-338-5p | Peli1 | 1223 | mmu-miR-423-5p | Supt6h |
| 333 | mmu-miR-338-5p | Rab28 | 1224 | mmu-miR-423-5p | Myo1d |
| 334 | mmu-miR-338-5p | Adamts16 | 1225 | mmu-miR-423-5p | N4bp2l1 |
| 335 | mmu-miR-338-5p | Senp7 | 1226 | mmu-miR-423-5p | Sp6 |
| 336 | mmu-miR-338-5p | Hmg20a | 1227 | mmu-miR-423-5p | Lasp1 |
| 337 | mmu-miR-338-5p | Cd38 | 1228 | mmu-miR-423-5p | Mlx |
| 338 | mmu-miR-338-5p | Ldb2 | 1229 | mmu-miR-423-5p | Psme3 |
| 339 | mmu-miR-338-5p | Itgb2 | 1230 | mmu-miR-423-5p | Entpd6 |
| 340 | mmu-miR-338-5p | Aak1 | 1231 | mmu-miR-423-5p | Sox12 |
| 341 | mmu-miR-338-5p | Slit2 | 1232 | mmu-miR-423-5p | Wipi1 |
| 342 | mmu-miR-338-5p | Cul3 | 1233 | mmu-miR-423-5p | Slc39a11 |
| 343 | mmu-miR-338-5p | Erap1 | 1234 | mmu-miR-423-5p | Map1lc3a |
| 344 | mmu-miR-338-5p | Nell2 | 1235 | mmu-miR-423-5p | Acss2 |
| 345 | mmu-miR-338-5p | Tshz3 | 1236 | mmu-miR-423-5p | Mybl2 |
| 346 | mmu-miR-338-5p | Col4a3 | 1237 | mmu-miR-423-5p | Rbm38 |
| 347 | mmu-miR-338-5p | Gata2 | 1238 | mmu-miR-423-5p | Chrna4 |
| 348 | mmu-miR-338-5p | Chac2 | 1239 | mmu-miR-429 | Rap1b |
| 349 | mmu-miR-338-5p | Ptma | 1240 | mmu-miR-429 | Rnd3 |
| 350 | mmu-miR-338-5p | Hormad1 | 1241 | mmu-miR-429 | Zeb1 |
| 351 | mmu-miR-338-5p | Slk | 1242 | mmu-miR-429 | Zfpm2 |
| 352 | mmu-miR-338-5p | Ccdc122 | 1243 | mmu-miR-429 | Npc1 |
| 353 | mmu-miR-338-5p | Cirbp | 1244 | mmu-miR-429 | Mboat2 |
| 354 | mmu-miR-338-5p | Gnpda2 | 1245 | mmu-miR-429 | Sulf1 |
| 355 | mmu-miR-338-5p | Frmd4b | 1246 | mmu-miR-429 | Rdh10 |
| 356 | mmu-miR-338-5p | Narg1l | 1247 | mmu-miR-429 | Cthrc1 |
| 357 | mmu-miR-338-5p | Dmd | 1248 | mmu-miR-429 | Cited2 |
| 358 | mmu-miR-338-5p | Dazap1 | 1249 | mmu-miR-429 | Matr3 |
| 359 | mmu-miR-338-5p | Slit3 | 1250 | mmu-miR-429 | Eapp |
| 360 | mmu-miR-338-5p | Hsdl2 | 1251 | mmu-miR-429 | Sec23a |
| 361 | mmu-miR-338-5p | Neto2 | 1252 | mmu-miR-429 | Wapal |
| 362 | mmu-miR-338-5p | Pcdh20 | 1253 | mmu-miR-429 | Sesn1 |
| 363 | mmu-miR-338-5p | Cxcr7 | 1254 | mmu-miR-429 | Dusp1 |
| 364 | mmu-miR-338-5p | Zfx | 1255 | mmu-miR-429 | Tmeff2 |
| 365 | mmu-miR-338-5p | Runx1 | 1256 | mmu-miR-429 | Nup153 |
| 366 | mmu-miR-338-5p | Zxdb | 1257 | mmu-miR-429 | Gpm6a |
| 367 | mmu-miR-338-5p | Spin4 | 1258 | mmu-miR-429 | Ptpn21 |
| 368 | mmu-miR-338-5p | Fbxl3 | 1259 | mmu-miR-429 | C030046E11Rik |
| 369 | mmu-miR-338-5p | Acvr1b | 1260 | mmu-miR-429 | Lats2 |
| 370 | mmu-miR-338-5p | Tnpo1 | 1261 | mmu-miR-429 | Tbc1d12 |
| 371 | mmu-miR-338-5p | Rnf145 | 1262 | mmu-miR-429 | Bag5 |
| 372 | mmu-miR-338-5p | Rnf219 | 1263 | mmu-miR-429 | Slit2 |
| 373 | mmu-miR-338-5p | Atrnl1 | 1264 | mmu-miR-429 | Hrb |
| 374 | mmu-miR-338-5p | Ets2 | 1265 | mmu-miR-429 | Slc38a2 |
| 375 | mmu-miR-338-5p | Bnip2 | 1266 | mmu-miR-429 | Fbxw11 |
| 376 | mmu-miR-338-5p | Gcnt3 | 1267 | mmu-miR-429 | Frmd4b |
| 377 | mmu-miR-338-5p | St8sia4 | 1268 | mmu-miR-429 | Lin7b |
| 378 | mmu-miR-338-5p | Rnf111 | 1269 | mmu-miR-429 | Amfr |
| 379 | mmu-miR-338-5p | Pappa | 1270 | mmu-miR-429 | Slc6a1 |
| 380 | mmu-miR-338-5p | Pparg | 1271 | mmu-miR-429 | Cdh11 |
| 381 | mmu-miR-338-5p | Zic2 | 1272 | mmu-miR-429 | Fhod1 |
| 382 | mmu-miR-338-5p | Cenpk | 1273 | mmu-miR-429 | Ntf3 |
| 383 | mmu-miR-338-5p | Zmym3 | 1274 | mmu-miR-429 | Rab21 |
| 384 | mmu-miR-338-5p | Dclre1b | 1275 | mmu-miR-429 | Wif1 |
| 385 | mmu-miR-338-5p | Ythdc1 | 1276 | mmu-miR-429 | Fxr2 |
| 386 | mmu-miR-338-5p | Ccdc102a | 1277 | mmu-miR-429 | Rab6ip1 |
| 387 | mmu-miR-338-5p | Rsbn1 | 1278 | mmu-miR-429 | Crtap |
| 388 | mmu-miR-338-5p | Slc17a6 | 1279 | mmu-miR-429 | Hoxd10 |
| 389 | mmu-miR-338-5p | Galnact2 | 1280 | mmu-miR-429 | Esrrg |
| 390 | mmu-miR-338-5p | Unc13c | 1281 | mmu-miR-429 | Zc3h15 |
| 391 | mmu-miR-338-5p | Daam2 | 1282 | mmu-miR-429 | Pds5b |
| 392 | mmu-miR-338-5p | Tubgcp5 | 1283 | mmu-miR-429 | Hlf |
| 393 | mmu-miR-338-5p | Dazl | 1284 | mmu-miR-429 | Errfi1 |
| 394 | mmu-miR-338-5p | A230046K03Rik | 1285 | mmu-miR-429 | Mapre1 |
| 395 | mmu-miR-338-5p | Kat2b | 1286 | mmu-miR-429 | Cbx4 |
| 396 | mmu-miR-338-5p | Sec24a | 1287 | mmu-miR-455 | Colec12 |
| 397 | mmu-miR-338-5p | Slc5a7 | 1288 | mmu-miR-455 | Esco1 |
| 398 | mmu-miR-338-5p | Ttc39b | 1289 | mmu-miR-455 | Lpin1 |
| 399 | mmu-miR-338-5p | Cdkl2 | 1290 | mmu-miR-455 | Grhl1 |
| 400 | mmu-miR-338-5p | Ccnt2 | 1291 | mmu-miR-455 | Evi5l |
| 401 | mmu-miR-338-5p | Jmjd2b | 1292 | mmu-miR-455 | Nol4 |
| 402 | mmu-miR-338-5p | Gnptab | 1293 | mmu-miR-455 | Ppp1r9a |
| 403 | mmu-miR-338-5p | Tmem30a | 1294 | mmu-miR-455 | Snn |
| 404 | mmu-miR-338-5p | Zfp711 | 1295 | mmu-miR-455 | Has1 |
| 405 | mmu-miR-338-5p | Irak1bp1 | 1296 | mmu-miR-455 | 2810012G03Rik |
| 406 | mmu-miR-338-5p | Acsl6 | 1297 | mmu-miR-455 | Hbegf |
| 407 | mmu-miR-338-5p | Mrpl1 | 1298 | mmu-miR-455 | Btn2a2 |
| 408 | mmu-miR-338-5p | Nap1l3 | 1299 | mmu-miR-455 | Stx7 |
| 409 | mmu-miR-338-5p | Nudt12 | 1300 | mmu-miR-455 | Dpy19l1 |
| 410 | mmu-miR-338-5p | Ankrd12 | 1301 | mmu-miR-455 | Zfp474 |
| 411 | mmu-miR-338-5p | AC171108.2 | 1302 | mmu-miR-455 | Snx2 |
| 412 | mmu-miR-338-5p | Eea1 | 1303 | mmu-miR-455 | Ufm1 |
| 413 | mmu-miR-338-5p | Dusp6 | 1304 | mmu-miR-455 | Mfsd9 |
| 414 | mmu-miR-338-5p | Kitl | 1305 | mmu-miR-455 | Slc12a2 |
| 415 | mmu-miR-338-5p | 2610101N10Rik | 1306 | mmu-miR-455 | Ssr1 |
| 416 | mmu-miR-338-5p | Hdgfrp3 | 1307 | mmu-miR-455 | Nup153 |
| 417 | mmu-miR-338-5p | D3Bwg0562e | 1308 | mmu-miR-455 | BC035537 |
| 418 | mmu-miR-338-5p | Zbtb43 | 1309 | mmu-miR-455 | Ift80 |
| 419 | mmu-miR-338-5p | Bex2 | 1310 | mmu-miR-455 | Odz3 |
| 420 | mmu-miR-338-5p | Cd9 | 1311 | mmu-miR-455 | Dnajb12 |
| 421 | mmu-miR-338-5p | Dnajc6 | 1312 | mmu-miR-455 | Cdkn1a |
| 422 | mmu-miR-338-5p | Ntf3 | 1313 | mmu-miR-455 | Topors |
| 423 | mmu-miR-338-5p | Myf5 | 1314 | mmu-miR-455 | Otub2 |
| 424 | mmu-miR-338-5p | Abcd3 | 1315 | mmu-miR-455 | Cbln2 |
| 425 | mmu-miR-338-5p | Pde4b | 1316 | mmu-miR-455 | Mtmr1 |
| 426 | mmu-miR-338-5p | Tmem135 | 1317 | mmu-miR-455 | Dip2a |
| 427 | mmu-miR-338-5p | Osbpl8 | 1318 | mmu-miR-455 | Cblb |
| 428 | mmu-miR-338-5p | Sec24d | 1319 | mmu-miR-455 | Imp3 |
| 429 | mmu-miR-338-5p | Ppap2b | 1320 | mmu-miR-455 | Ptpn9 |
| 430 | mmu-miR-338-5p | Mtf2 | 1321 | mmu-miR-455 | Tmem34 |
| 431 | mmu-miR-338-5p | Ltbp1 | 1322 | mmu-miR-455 | Pnma2 |
| 432 | mmu-miR-338-5p | Amotl2 | 1323 | mmu-miR-455 | Hif1an |
| 433 | mmu-miR-338-5p | Rab6b | 1324 | mmu-miR-455 | Cul3 |
| 434 | mmu-miR-338-5p | Ptgs1 | 1325 | mmu-miR-455 | Cyp1a1 |
| 435 | mmu-miR-338-5p | Sfrs7 | 1326 | mmu-miR-455 | Scoc |
| 436 | mmu-miR-338-5p | Papss1 | 1327 | mmu-miR-455 | Rtn4 |
| 437 | mmu-miR-338-5p | Atp2c1 | 1328 | mmu-miR-455 | Taz |
| 438 | mmu-miR-338-5p | Wif1 | 1329 | mmu-miR-455 | Rhbdf1 |
| 439 | mmu-miR-338-5p | Pgm2l1 | 1330 | mmu-miR-455 | Sucla2 |
| 440 | mmu-miR-338-5p | Zcchc11 | 1331 | mmu-miR-455 | Kbtbd7 |
| 441 | mmu-miR-338-5p | Zeb2 | 1332 | mmu-miR-455 | Dnaja2 |
| 442 | mmu-miR-338-5p | Acvr2a | 1333 | mmu-miR-455 | Sh3bp4 |
| 443 | mmu-miR-338-5p | Rgs16 | 1334 | mmu-miR-455 | Prkab2 |
| 444 | mmu-miR-338-5p | Mbd5 | 1335 | mmu-miR-455 | Csnk1g1 |
| 445 | mmu-miR-338-5p | Lypd6 | 1336 | mmu-miR-455 | AC109199.10 |
| 446 | mmu-miR-338-5p | Rnd3 | 1337 | mmu-miR-455 | Dscr3 |
| 447 | mmu-miR-338-5p | Dock3 | 1338 | mmu-miR-455 | Rbm26 |
| 448 | mmu-miR-338-5p | Fbxo11 | 1339 | mmu-miR-455 | Hoxc4 |
| 449 | mmu-miR-338-5p | Bmpr1b | 1340 | mmu-miR-455 | Phka1 |
| 450 | mmu-miR-338-5p | Adcyap1 | 1341 | mmu-miR-455 | Luzp2 |
| 451 | mmu-miR-338-5p | Cacnb4 | 1342 | mmu-miR-455 | Mobkl1a |
| 452 | mmu-miR-338-5p | Fmnl2 | 1343 | mmu-miR-455 | Map3k1 |
| 453 | mmu-miR-338-5p | Mageh1 | 1344 | mmu-miR-455 | Nuak1 |
| 454 | mmu-miR-338-5p | Rragb | 1345 | mmu-miR-455 | Tor1b |
| 455 | mmu-miR-338-5p | Lrp1 | 1346 | mmu-miR-455 | Ndn |
| 456 | mmu-miR-338-5p | 4732418C07Rik | 1347 | mmu-miR-455 | Itga1 |
| 457 | mmu-miR-338-5p | Col24a1 | 1348 | mmu-miR-455 | Zcchc5 |
| 458 | mmu-miR-338-5p | Baz2b | 1349 | mmu-miR-455 | Myo6 |
| 459 | mmu-miR-338-5p | Klhdc5 | 1350 | mmu-miR-455 | Jun |
| 460 | mmu-miR-338-5p | Blzf1 | 1351 | mmu-miR-455 | Kcne1l |
| 461 | mmu-miR-338-5p | Pigk | 1352 | mmu-miR-455 | Dio1 |
| 462 | mmu-miR-338-5p | Reps2 | 1353 | mmu-miR-455 | Usp10 |
| 463 | mmu-miR-338-5p | Ap1s2 | 1354 | mmu-miR-455 | H2afz |
| 464 | mmu-miR-338-5p | Scn9a | 1355 | mmu-miR-455 | Sh3glb1 |
| 465 | mmu-miR-338-5p | Depdc1a | 1356 | mmu-miR-455 | Ptges3 |
| 466 | mmu-miR-338-5p | Gpm6b | 1357 | mmu-miR-455 | Bicd1 |
| 467 | mmu-miR-338-5p | Lrp2 | 1358 | mmu-miR-455 | Cmc1 |
| 468 | mmu-miR-338-5p | Bbs5 | 1359 | mmu-miR-455 | Zfp238 |
| 469 | mmu-miR-338-5p | Adm | 1360 | mmu-miR-455 | Sccpdh |
| 470 | mmu-miR-338-5p | Nlgn2 | 1361 | mmu-miR-455 | Pafah1b1 |
| 471 | mmu-miR-338-5p | Foxj3 | 1362 | mmu-miR-455 | Madd |
| 472 | mmu-miR-338-5p | Dync1i2 | 1363 | mmu-miR-455 | Poldip2 |
| 473 | mmu-miR-338-5p | Slc25a12 | 1364 | mmu-miR-455 | Fbxo42 |
| 474 | mmu-miR-338-5p | Calca | 1365 | mmu-miR-455 | Cltc |
| 475 | mmu-miR-338-5p | Sp3 | 1366 | mmu-miR-455 | Bmf |
| 476 | mmu-miR-338-5p | Aim2 | 1367 | mmu-miR-455 | Hlf |
| 477 | mmu-miR-338-5p | Wipf1 | 1368 | mmu-miR-455 | Cntd1 |
| 478 | mmu-miR-338-5p | Tmem159 | 1369 | mmu-miR-455 | Gjc1 |
| 479 | mmu-miR-338-5p | Mtf1 | 1370 | mmu-miR-455 | Uqcc |
| 480 | mmu-miR-338-5p | Plekha3 | 1371 | mmu-miR-455 | Slc1a5 |
| 481 | mmu-miR-338-5p | Mark1 | 1372 | mmu-miR-455 | Sar1a |
| 482 | mmu-miR-338-5p | Zzef1 | 1373 | mmu-miR-455 | Amica1 |
| 483 | mmu-miR-338-5p | Esrrg | 1374 | mmu-miR-455 | Odz4 |
| 484 | mmu-miR-338-5p | Vkorc1l1 | 1375 | mmu-miR-455 | 2610207I05Rik |
| 485 | mmu-miR-338-5p | Ppp2r5a | 1376 | mmu-miR-497 | Pcmt1 |
| 486 | mmu-miR-338-5p | A130010J15Rik | 1377 | mmu-miR-497 | Abhd13 |
| 487 | mmu-miR-338-5p | Mnt | 1378 | mmu-miR-497 | Ccdc28a |
| 488 | mmu-miR-338-5p | Pum1 | 1379 | mmu-miR-497 | Tcfap2d |
| 489 | mmu-miR-338-5p | Cldn15 | 1380 | mmu-miR-497 | Arl2 |
| 490 | mmu-miR-338-5p | Nufip2 | 1381 | mmu-miR-497 | Sil1 |
| 491 | mmu-miR-338-5p | Clic4 | 1382 | mmu-miR-497 | Sgk1 |
| 492 | mmu-miR-338-5p | Creb3l1 | 1383 | mmu-miR-497 | Carm1 |
| 493 | mmu-miR-338-5p | Nf1 | 1384 | mmu-miR-497 | Dmtf1 |
| 494 | mmu-miR-338-5p | Pdhx | 1385 | mmu-miR-497 | Sema3a |
| 495 | mmu-miR-338-5p | Abtb2 | 1386 | mmu-miR-497 | Hspa4l |
| 496 | mmu-miR-338-5p | Med13 | 1387 | mmu-miR-497 | Hgf |
| 497 | mmu-miR-338-5p | Cltc | 1388 | mmu-miR-497 | Fnta |
| 498 | mmu-miR-338-5p | Pan3 | 1389 | mmu-miR-497 | Wapal |
| 499 | mmu-miR-338-5p | Ubl3 | 1390 | mmu-miR-497 | Srpr |
| 500 | mmu-miR-338-5p | Zfp770 | 1391 | mmu-miR-497 | Wwp1 |
| 501 | mmu-miR-338-5p | Tom1l1 | 1392 | mmu-miR-497 | Gcc2 |
| 502 | mmu-miR-338-5p | Tob1 | 1393 | mmu-miR-497 | Gpr63 |
| 503 | mmu-miR-338-5p | Zfp106 | 1394 | mmu-miR-497 | Eif2b2 |
| 504 | mmu-miR-338-5p | Errfi1 | 1395 | mmu-miR-497 | Plrg1 |
| 505 | mmu-miR-338-5p | Ccndbp1 | 1396 | mmu-miR-497 | Hoxa10 |
| 506 | mmu-miR-338-5p | Sp2 | 1397 | mmu-miR-497 | Cdc37l1 |
| 507 | mmu-miR-338-5p | P140 | 1398 | mmu-miR-497 | Smad7 |
| 508 | mmu-miR-338-5p | Ctdspl2 | 1399 | mmu-miR-497 | Rabl3 |
| 509 | mmu-miR-338-5p | Fgf7 | 1400 | mmu-miR-497 | AC122398.2 |
| 510 | mmu-miR-338-5p | Nr1d1 | 1401 | mmu-miR-497 | Lats2 |
| 511 | mmu-miR-338-5p | Ciao1 | 1402 | mmu-miR-497 | Setd3 |
| 512 | mmu-miR-338-5p | Kif16b | 1403 | mmu-miR-497 | Pou4f2 |
| 513 | mmu-miR-338-5p | Nsf | 1404 | mmu-miR-497 | Pepd |
| 514 | mmu-miR-338-5p | Nanp | 1405 | mmu-miR-497 | Rad23b |
| 515 | mmu-miR-338-5p | Id1 | 1406 | mmu-miR-497 | Nup210 |
| 516 | mmu-miR-338-5p | Ddx5 | 1407 | mmu-miR-497 | Taf5 |
| 517 | mmu-miR-338-5p | Ccdc46 | 1408 | mmu-miR-497 | Lhx3 |
| 518 | mmu-miR-338-5p | Gaa | 1409 | mmu-miR-497 | Ap3b1 |
| 519 | mmu-miR-338-5p | Ncoa3 | 1410 | mmu-miR-497 | Shoc2 |
| 520 | mmu-miR-338-5p | Snai1 | 1411 | mmu-miR-497 | Ap3d1 |
| 521 | mmu-miR-338-5p | Foxk2 | 1412 | mmu-miR-497 | Eif3a |
| 522 | mmu-miR-338-5p | Tmepai | 1413 | mmu-miR-497 | Ppap2a |
| 523 | mmu-miR-338-5p | Gnas | 1414 | mmu-miR-497 | Prdm4 |
| 524 | mmu-miR-338-5p | Ss18l1 | 1415 | mmu-miR-497 | Kcnc4 |
| 525 | mmu-miR-338-5p | Ythdf1 | 1416 | mmu-miR-497 | Wbp11 |
| 526 | mmu-miR-338-5p | Arhgap18 | 1417 | mmu-miR-497 | Adh5 |
| 527 | mmu-miR-338-5p | 4921505C17Rik | 1418 | mmu-miR-497 | Col24a1 |
| 528 | mmu-miR-338-5p | Tmem168 | 1419 | mmu-miR-497 | Rnf10 |
| 529 | mmu-miR-338-5p | Rad21 | 1420 | mmu-miR-497 | Gorasp2 |
| 530 | mmu-miR-338-5p | Atp11b | 1421 | mmu-miR-497 | Arpp21 |
| 531 | mmu-miR-338-5p | Mnx1 | 1422 | mmu-miR-497 | Atxn2 |
| 532 | mmu-miR-338-5p | Pak4 | 1423 | mmu-miR-497 | Spag7 |
| 533 | mmu-miR-338-5p | Pi4k2b | 1424 | mmu-miR-497 | Mapk3 |
| 534 | mmu-miR-338-5p | Atrx | 1425 | mmu-miR-497 | Slc6a4 |
| 535 | mmu-miR-340-3p | Ppp1cb | 1426 | mmu-miR-497 | Wsb1 |
| 536 | mmu-miR-340-3p | Speg | 1427 | mmu-miR-497 | Suz12 |
| 537 | mmu-miR-340-3p | Rgs1 | 1428 | mmu-miR-497 | Ap1gbp1 |
| 538 | mmu-miR-340-3p | Wdr42a | 1429 | mmu-miR-497 | Ppm1d |
| 539 | mmu-miR-340-3p | Atxn2l | 1430 | mmu-miR-497 | Cox11 |
| 540 | mmu-miR-340-3p | Blcap | 1431 | mmu-miR-497 | Tasp1 |
| 541 | mmu-miR-340-3p | Rbj | 1432 | mmu-miR-497 | Fkbp1a |
| 542 | mmu-miR-340-3p | Trib2 | 1433 | mmu-miR-497 | Mapre1 |
| 543 | mmu-miR-340-3p | Impact | 1434 | mmu-miR-497 | Dhx35 |
| 544 | mmu-miR-340-3p | Rps6ka2 | 1435 | mmu-miR-497 | Cbx4 |
| 545 | mmu-miR-340-3p | Rnf125 | 1436 | mmu-miR-497 | Fasn |
| 546 | mmu-miR-340-3p | T | 1437 | mmu-miR-497 | Slc20a2 |
| 547 | mmu-miR-340-3p | BC052328 | 1438 | mmu-miR-497 | Med26 |
| 548 | mmu-miR-340-3p | Snapc2 | 1439 | mmu-miR-497 | Btrc |
| 549 | mmu-miR-340-3p | Nid2 | 1440 | mmu-miR-497 | Cops7b |
| 550 | mmu-miR-340-3p | Amotl1 | 1441 | mmu-miR-497 | C1qtnf2 |
| 551 | mmu-miR-340-3p | Amph | 1442 | mmu-miR-497 | Calcrl |
| 552 | mmu-miR-340-3p | Tmco3 | 1443 | mmu-miR-532-5p | Myct1 |
| 553 | mmu-miR-340-3p | Batf2 | 1444 | mmu-miR-532-5p | Gm944 |
| 554 | mmu-miR-340-3p | Il17rd | 1445 | mmu-miR-532-5p | Tcea1 |
| 555 | mmu-miR-340-3p | Wasl | 1446 | mmu-miR-532-5p | Lrp11 |
| 556 | mmu-miR-340-3p | Cacna1d | 1447 | mmu-miR-532-5p | Pcmtd1 |
| 557 | mmu-miR-340-3p | Nkx2-1 | 1448 | mmu-miR-532-5p | Zfp704 |
| 558 | mmu-miR-340-3p | Trib1 | 1449 | mmu-miR-532-5p | Abat |
| 559 | mmu-miR-340-3p | Crkl | 1450 | mmu-miR-532-5p | Slc25a46 |
| 560 | mmu-miR-340-3p | Ube2h | 1451 | mmu-miR-532-5p | Snx16 |
| 561 | mmu-miR-340-3p | Myst3 | 1452 | mmu-miR-532-5p | Rnf19a |
| 562 | mmu-miR-340-3p | Spry4 | 1453 | mmu-miR-532-5p | Camk2g |
| 563 | mmu-miR-340-3p | Sfrp1 | 1454 | mmu-miR-532-5p | Vcl |
| 564 | mmu-miR-340-3p | Tacc1 | 1455 | mmu-miR-532-5p | Clec16a |
| 565 | mmu-miR-340-3p | Tspan7 | 1456 | mmu-miR-532-5p | Tnfsf10 |
| 566 | mmu-miR-340-3p | Wdr91 | 1457 | mmu-miR-532-5p | Il17rd |
| 567 | mmu-miR-340-3p | Hira | 1458 | mmu-miR-532-5p | Srrm2 |
| 568 | mmu-miR-340-3p | Klhl24 | 1459 | mmu-miR-532-5p | Chchd3 |
| 569 | mmu-miR-340-3p | Tnfaip8 | 1460 | mmu-miR-532-5p | Slc25a31 |
| 570 | mmu-miR-340-3p | Ets1 | 1461 | mmu-miR-532-5p | Prkch |
| 571 | mmu-miR-340-3p | Ndph | 1462 | mmu-miR-532-5p | Bmpr1a |
| 572 | mmu-miR-340-3p | Spg20 | 1463 | mmu-miR-532-5p | Cdc2l6 |
| 573 | mmu-miR-340-3p | Ccdc86 | 1464 | mmu-miR-532-5p | Rlbp1l1 |
| 574 | mmu-miR-340-3p | Max | 1465 | mmu-miR-532-5p | Ms4a15 |
| 575 | mmu-miR-340-3p | Stub1 | 1466 | mmu-miR-532-5p | Dclk1 |
| 576 | mmu-miR-340-3p | Wdr22 | 1467 | mmu-miR-532-5p | Tmem8 |
| 577 | mmu-miR-340-3p | Sobp | 1468 | mmu-miR-532-5p | Col5a2 |
| 578 | mmu-miR-340-3p | Camk2a | 1469 | mmu-miR-532-5p | Sucnr1 |
| 579 | mmu-miR-340-3p | Bak1 | 1470 | mmu-miR-532-5p | Atxn1 |
| 580 | mmu-miR-340-3p | BC035537 | 1471 | mmu-miR-532-5p | Opa1 |
| 581 | mmu-miR-340-3p | Napg | 1472 | mmu-miR-532-5p | Stk38 |
| 582 | mmu-miR-340-3p | Rnf185 | 1473 | mmu-miR-532-5p | Mtap9 |
| 583 | mmu-miR-340-3p | Zfp202 | 1474 | mmu-miR-532-5p | Pgm5 |
| 584 | mmu-miR-340-3p | Kpna4 | 1475 | mmu-miR-532-5p | Akap8 |
| 585 | mmu-miR-340-3p | Osbp2 | 1476 | mmu-miR-532-5p | Yes1 |
| 586 | mmu-miR-340-3p | Zfp775 | 1477 | mmu-miR-532-5p | Papss2 |
| 587 | mmu-miR-340-3p | Bicd2 | 1478 | mmu-miR-532-5p | Pram1 |
| 588 | mmu-miR-340-3p | Irf2 | 1479 | mmu-miR-532-5p | Rpe |
| 589 | mmu-miR-340-3p | Sgol2 | 1480 | mmu-miR-532-5p | Cpeb3 |
| 590 | mmu-miR-340-3p | Rps6kl1 | 1481 | mmu-miR-532-5p | Adcy1 |
| 591 | mmu-miR-340-3p | Grik5 | 1482 | mmu-miR-532-5p | Lrriq2 |
| 592 | mmu-miR-340-3p | Impa2 | 1483 | mmu-miR-532-5p | Hs3st1 |
| 593 | mmu-miR-340-3p | Elfn2 | 1484 | mmu-miR-532-5p | Aldh1b1 |
| 594 | mmu-miR-340-3p | Oaf | 1485 | mmu-miR-532-5p | Abcd2 |
| 595 | mmu-miR-340-3p | Tmem145 | 1486 | mmu-miR-532-5p | Epha4 |
| 596 | mmu-miR-340-3p | Pvrl1 | 1487 | mmu-miR-532-5p | Adarb1 |
| 597 | mmu-miR-340-3p | Gucy1b3 | 1488 | mmu-miR-532-5p | Rhobtb3 |
| 598 | mmu-miR-340-3p | Cplx2 | 1489 | mmu-miR-532-5p | Senp8 |
| 599 | mmu-miR-340-3p | Faf2 | 1490 | mmu-miR-532-5p | Pde6d |
| 600 | mmu-miR-340-3p | Unc5b | 1491 | mmu-miR-532-5p | Bach1 |
| 601 | mmu-miR-340-3p | Ocrl | 1492 | mmu-miR-532-5p | Ubac1 |
| 602 | mmu-miR-340-3p | Nod1 | 1493 | mmu-miR-532-5p | Pls3 |
| 603 | mmu-miR-340-3p | Myo5b | 1494 | mmu-miR-532-5p | Sorcs3 |
| 604 | mmu-miR-340-3p | Ptpn21 | 1495 | mmu-miR-532-5p | Atp8a1 |
| 605 | mmu-miR-340-3p | Mll1 | 1496 | mmu-miR-532-5p | Kctd8 |
| 606 | mmu-miR-340-3p | Fcrl1 | 1497 | mmu-miR-532-5p | Pcdh20 |
| 607 | mmu-miR-340-3p | Amica1 | 1498 | mmu-miR-532-5p | Pcdh9 |
| 608 | mmu-miR-340-3p | Sgms1 | 1499 | mmu-miR-532-5p | Zxdb |
| 609 | mmu-miR-340-3p | Pten | 1500 | mmu-miR-532-5p | Clint1 |
| 610 | mmu-miR-340-3p | Gmip | 1501 | mmu-miR-532-5p | Mtap1b |
| 611 | mmu-miR-340-3p | Setbp1 | 1502 | mmu-miR-532-5p | Tpm1 |
| 612 | mmu-miR-340-3p | Mad2l1 | 1503 | mmu-miR-532-5p | Chd9 |
| 613 | mmu-miR-340-3p | Ifit2 | 1504 | mmu-miR-532-5p | Yipf6 |
| 614 | mmu-miR-340-3p | Tshz1 | 1505 | mmu-miR-532-5p | Kcnc1 |
| 615 | mmu-miR-340-3p | Rmnd5a | 1506 | mmu-miR-532-5p | Serpinb13 |
| 616 | mmu-miR-340-3p | AC145168.5 | 1507 | mmu-miR-532-5p | Galnact2 |
| 617 | mmu-miR-340-3p | Cpeb3 | 1508 | mmu-miR-532-5p | Csf1 |
| 618 | mmu-miR-340-3p | Pacsin2 | 1509 | mmu-miR-532-5p | Lpar4 |
| 619 | mmu-miR-340-3p | Dicer1 | 1510 | mmu-miR-532-5p | Slc5a8 |
| 620 | mmu-miR-340-3p | Tfam | 1511 | mmu-miR-532-5p | Chm |
| 621 | mmu-miR-340-3p | Ash1l | 1512 | mmu-miR-532-5p | Rlbp1 |
| 622 | mmu-miR-340-3p | Sidt1 | 1513 | mmu-miR-532-5p | Nt5e |
| 623 | mmu-miR-340-3p | Pou2af1 | 1514 | mmu-miR-532-5p | Plod2 |
| 624 | mmu-miR-340-3p | Efna4 | 1515 | mmu-miR-532-5p | Raver2 |
| 625 | mmu-miR-340-3p | Thnsl1 | 1516 | mmu-miR-532-5p | Frs2 |
| 626 | mmu-miR-340-3p | Il6ra | 1517 | mmu-miR-532-5p | Wfdc1 |
| 627 | mmu-miR-340-3p | Mapk12 | 1518 | mmu-miR-532-5p | Traip |
| 628 | mmu-miR-340-3p | Atp8b2 | 1519 | mmu-miR-532-5p | Acvr1c |
| 629 | mmu-miR-340-3p | Peli1 | 1520 | mmu-miR-532-5p | Prrx1 |
| 630 | mmu-miR-340-3p | Rab13 | 1521 | mmu-miR-532-5p | Rbbp7 |
| 631 | mmu-miR-340-3p | Spopl | 1522 | mmu-miR-532-5p | Fbxl2 |
| 632 | mmu-miR-340-3p | Chkb | 1523 | mmu-miR-532-5p | Sestd1 |
| 633 | mmu-miR-340-3p | Loxl4 | 1524 | mmu-miR-532-5p | Oat |
| 634 | mmu-miR-340-3p | Ccdc25 | 1525 | mmu-miR-532-5p | Ppp2r2d |
| 635 | mmu-miR-340-3p | Aldh1b1 | 1526 | mmu-miR-532-5p | Hoxb3 |
| 636 | mmu-miR-340-3p | Hmg20a | 1527 | mmu-miR-532-5p | Dffb |
| 637 | mmu-miR-340-3p | Tmod1 | 1528 | mmu-miR-532-5p | Socs7 |
| 638 | mmu-miR-340-3p | Chst8 | 1529 | mmu-miR-532-5p | Ctdspl2 |
| 639 | mmu-miR-340-3p | Cebpg | 1530 | mmu-miR-532-5p | Gatm |
| 640 | mmu-miR-340-3p | Glt8d3 | 1531 | mmu-miR-532-5p | Pak7 |
| 641 | mmu-miR-340-3p | Dock5 | 1532 | mmu-miR-532-5p | Pcdha10 |
| 642 | mmu-miR-340-3p | Scd1 | 1533 | mmu-miR-532-5p | Kcnab1 |
| 643 | mmu-miR-340-3p | Nfkbil1 | 1534 | mmu-miR-669d | Insr |
| 644 | mmu-miR-340-3p | Tdrkh | 1535 | mmu-miR-669d | Gria4 |
| 645 | mmu-miR-340-3p | Tmeff1 | 1536 | mmu-miR-669d | Syne1 |
| 646 | mmu-miR-340-3p | Ruvbl1 | 1537 | mmu-miR-669d | Bet1 |
| 647 | mmu-miR-340-3p | Ldb1 | 1538 | mmu-miR-669d | Sox11 |
| 648 | mmu-miR-340-3p | Vdr | 1539 | mmu-miR-669d | Syt4 |
| 649 | mmu-miR-340-3p | Nrm | 1540 | mmu-miR-669d | Txlnb |
| 650 | mmu-miR-340-3p | Brunol6 | 1541 | mmu-miR-669d | Snai2 |
| 651 | mmu-miR-340-3p | Klk9 | 1542 | mmu-miR-669d | Slc25a21 |
| 652 | mmu-miR-340-3p | Gpr55 | 1543 | mmu-miR-669d | Ndufb5 |
| 653 | mmu-miR-340-3p | Rnd1 | 1544 | mmu-miR-669d | Nsmce2 |
| 654 | mmu-miR-340-3p | Tmem43 | 1545 | mmu-miR-669d | Lancl3 |
| 655 | mmu-miR-340-3p | Phox2b | 1546 | mmu-miR-669d | Kcnj5 |
| 656 | mmu-miR-340-3p | Slc30a9 | 1547 | mmu-miR-669d | Maob |
| 657 | mmu-miR-340-3p | Smad3 | 1548 | mmu-miR-669d | Nrn1 |
| 658 | mmu-miR-340-3p | Jmy | 1549 | mmu-miR-669d | Gcm2 |
| 659 | mmu-miR-340-3p | Midn | 1550 | mmu-miR-669d | Kcnab1 |
| 660 | mmu-miR-340-3p | Tegt | 1551 | mmu-miR-669d | Lpp |
| 661 | mmu-miR-340-3p | Aqp2 | 1552 | mmu-miR-669d | Pik3ip1 |
| 662 | mmu-miR-340-3p | Mxi1 | 1553 | mmu-miR-669d | Uhrf1bp1 |
| 663 | mmu-miR-340-3p | Notch1 | 1554 | mmu-miR-669d | Rwdd4a |
| 664 | mmu-miR-340-3p | Lima1 | 1555 | mmu-miR-669d | Batf |
| 665 | mmu-miR-340-3p | Centg2 | 1556 | mmu-miR-669d | Sh2d1a |
| 666 | mmu-miR-340-3p | Tcfe2a | 1557 | mmu-miR-669d | Zfand3 |
| 667 | mmu-miR-340-3p | Gabra1 | 1558 | mmu-miR-669d | Tax1bp1 |
| 668 | mmu-miR-340-3p | Usp46 | 1559 | mmu-miR-669d | Sel1l |
| 669 | mmu-miR-340-3p | Tbx15 | 1560 | mmu-miR-669d | Smad7 |
| 670 | mmu-miR-340-3p | Rnf183 | 1561 | mmu-miR-669d | Ktelc1 |
| 671 | mmu-miR-340-3p | Clock | 1562 | mmu-miR-669d | Pou2af1 |
| 672 | mmu-miR-340-3p | Hspa12a | 1563 | mmu-miR-669d | Bnip3l |
| 673 | mmu-miR-340-3p | Hdlbp | 1564 | mmu-miR-669d | Lsm14a |
| 674 | mmu-miR-340-3p | Spink2 | 1565 | mmu-miR-669d | Kcnip4 |
| 675 | mmu-miR-340-3p | Atcay | 1566 | mmu-miR-669d | Tnf |
| 676 | mmu-miR-340-3p | 6430514L14Rik | 1567 | mmu-miR-669d | Aldob |
| 677 | mmu-miR-340-3p | Cnpy3 | 1568 | mmu-miR-669d | Frmd4b |
| 678 | mmu-miR-340-3p | Ubac2 | 1569 | mmu-miR-669d | Odz2 |
| 679 | mmu-miR-340-3p | Mapk9 | 1570 | mmu-miR-669d | Kcne1 |
| 680 | mmu-miR-340-3p | Plxnd1 | 1571 | mmu-miR-669d | Zxdb |
| 681 | mmu-miR-340-3p | Ogt | 1572 | mmu-miR-669d | Per2 |
| 682 | mmu-miR-340-3p | Nfib | 1573 | mmu-miR-669d | Foxd1 |
| 683 | mmu-miR-340-3p | Ptges | 1574 | mmu-miR-669d | Adra1b |
| 684 | mmu-miR-340-3p | Btbd11 | 1575 | mmu-miR-669d | Wrb |
| 685 | mmu-miR-340-3p | Ccdc93 | 1576 | mmu-miR-669d | Rnf152 |
| 686 | mmu-miR-340-3p | Ebi3 | 1577 | mmu-miR-669d | Tmprss11f |
| 687 | mmu-miR-340-3p | Lypd1 | 1578 | mmu-miR-669d | Luzp2 |
| 688 | mmu-miR-340-3p | Leap2 | 1579 | mmu-miR-669d | Mllt3 |
| 689 | mmu-miR-340-3p | Cxcl9 | 1580 | mmu-miR-669d | Slc41a1 |
| 690 | mmu-miR-340-3p | Aldh1a3 | 1581 | mmu-miR-669d | Cstf2 |
| 691 | mmu-miR-340-3p | Fubp3 | 1582 | mmu-miR-669d | Hdgfrp3 |
| 692 | mmu-miR-340-3p | Igf1r | 1583 | mmu-miR-669d | Paqr9 |
| 693 | mmu-miR-340-3p | Sv2b | 1584 | mmu-miR-669d | Osbpl8 |
| 694 | mmu-miR-340-3p | Vav3 | 1585 | mmu-miR-669d | Camsap1l1 |
| 695 | mmu-miR-340-3p | Ntng1 | 1586 | mmu-miR-669d | Clec9a |
| 696 | mmu-miR-340-3p | Vps4a | 1587 | mmu-miR-669d | Wwox |
| 697 | mmu-miR-340-3p | Tbc1d2b | 1588 | mmu-miR-669d | Dcx |
| 698 | mmu-miR-340-3p | Txnl4b | 1589 | mmu-miR-669d | Txndc12 |
| 699 | mmu-miR-340-3p | Arhgap28 | 1590 | mmu-miR-669d | Cish |
| 700 | mmu-miR-340-3p | Tgif1 | 1591 | mmu-miR-669d | Slc38a3 |
| 701 | mmu-miR-340-3p | Ccnd2 | 1592 | mmu-miR-669d | Scyl1bp1 |
| 702 | mmu-miR-340-3p | Spsb4 | 1593 | mmu-miR-669d | Slc25a20 |
| 703 | mmu-miR-340-3p | Phlda3 | 1594 | mmu-miR-669d | Ubp1 |
| 704 | mmu-miR-340-3p | Pawr | 1595 | mmu-miR-669d | Xkr8 |
| 705 | mmu-miR-340-3p | Tgfbr3 | 1596 | mmu-miR-669d | Zkscan1 |
| 706 | mmu-miR-340-3p | Fzd4 | 1597 | mmu-miR-669d | Syt13 |
| 707 | mmu-miR-340-3p | Crebzf | 1598 | mmu-miR-669d | Frap1 |
| 708 | mmu-miR-340-3p | Tmem126b | 1599 | mmu-miR-669d | Slc30a4 |
| 709 | mmu-miR-340-3p | Rab3ip | 1600 | mmu-miR-669d | Thbd |
| 710 | mmu-miR-340-3p | Galm | 1601 | mmu-miR-669d | Foxk2 |
| 711 | mmu-miR-340-3p | Golga1 | 1602 | mmu-miR-669d | Kcnj12 |
| 712 | mmu-miR-340-3p | Pgm2l1 | 1603 | mmu-miR-802 | Abhd3 |
| 713 | mmu-miR-340-3p | Ulk1 | 1604 | mmu-miR-802 | Matr3 |
| 714 | mmu-miR-340-3p | Slc39a8 | 1605 | mmu-miR-802 | Slc20a2 |
| 715 | mmu-miR-340-3p | Cox4nb | 1606 | mmu-miR-802 | Arf6 |
| 716 | mmu-miR-340-3p | Plekhb1 | 1607 | mmu-miR-802 | Ccdc100 |
| 717 | mmu-miR-340-3p | Rgs16 | 1608 | mmu-miR-802 | Tox |
| 718 | mmu-miR-340-3p | Kif5c | 1609 | mmu-miR-802 | Aldh1a1 |
| 719 | mmu-miR-340-3p | Zcchc14 | 1610 | mmu-miR-802 | Hprt1 |
| 720 | mmu-miR-340-3p | Tspan5 | 1611 | mmu-miR-802 | Scg2 |
| 721 | mmu-miR-340-3p | Zc3h18 | 1612 | mmu-miR-802 | Hrb |
| 722 | mmu-miR-340-3p | Sart3 | 1613 | mmu-miR-802 | Ugdh |
| 723 | mmu-miR-340-3p | Mbd6 | 1614 | mmu-miR-802 | Bbs2 |
| 724 | mmu-miR-340-3p | Pmp22 | 1615 | mmu-miR-802 | Sfrs12 |
| 725 | mmu-miR-340-3p | Ihpk1 | 1616 | mmu-miR-802 | Sdccag10 |
| 726 | mmu-miR-340-3p | Fgfr1op2 | 1617 | mmu-miR-802 | Cdh11 |
| 727 | mmu-miR-340-3p | Rab35 | 1618 | mmu-miR-802 | Dync1li2 |
| 728 | mmu-miR-340-3p | Slc19a2 | 1619 | mmu-miR-802 | Ppp2ca |
| 729 | mmu-miR-340-3p | AC073946.23-201 | 1620 | mmu-miR-802 | Lrrk1 |
| 730 | mmu-miR-340-3p | Pan2 | 1621 | mmu-miR-802 | Sec11a |
| 731 | mmu-miR-340-3p | St6galnac3 | 1622 | mmu-miR-802 | Birc6 |
| 732 | mmu-miR-340-3p | Per1 | 1623 | mmu-miR-802 | Dr1 |
| 733 | mmu-miR-340-3p | Tnni3k | 1624 | mmu-miR-802 | Ppp3ca |
| 734 | mmu-miR-340-3p | Grpr | 1625 | mmu-miR-802 | Tada1l |
| 735 | mmu-miR-340-3p | Jmjd3 | 1626 | mmu-miR-802 | 0610025P10Rik |
| 736 | mmu-miR-340-3p | Swap70 | 1627 | mmu-miR-802 | Hnf1b |
| 737 | mmu-miR-340-3p | Foxj3 | 1628 | mmu-miR-802 | Aqr |
| 738 | mmu-miR-340-3p | Cited4 | 1629 | mmu-miR-802 | Nfe2l1 |
| 739 | mmu-miR-340-3p | Pea15a | 1630 | mmu-miR-802 | Mkx |
| 740 | mmu-miR-340-3p | Atp1a2 | 1631 | mmu-miR-802 | Rhob |
| 741 | mmu-miR-340-3p | P2rx4 | 1632 | mmu-miR-802 | Pqlc3 |
| 742 | mmu-miR-340-3p | Bcl6b | 1633 | mmu-miR-802 | Zfp521 |
| 743 | mmu-miR-340-3p | Wipf1 | 1634 | mmu-miR-802 | Sub1 |
| 744 | mmu-miR-340-3p | Bcl7a | 1635 | mmu-miR-802 | Aasdhppt |
| 745 | mmu-miR-340-3p | Axud1 | 1636 | mmu-miR-802 | Pdgfd |
| 746 | mmu-miR-340-3p | Hs3st2 | 1637 | mmu-miR-802 | Nol4 |
| 747 | mmu-miR-340-3p | Pitpnm2 | 1638 | mmu-miR-802 | Zfhx4 |
| 748 | mmu-miR-340-3p | Gga2 | 1639 | mmu-miR-802 | Bcap29 |
| 749 | mmu-miR-340-3p | Ubfd1 | 1640 | mmu-miR-802 | Ankrd10 |
| 750 | mmu-miR-340-3p | Tmem158 | 1641 | mmu-miR-802 | Map3k2 |
| 751 | mmu-miR-340-3p | Smtnl2 | 1642 | mmu-miR-802 | Cyp7b1 |
| 752 | mmu-miR-340-3p | Ak2 | 1643 | mmu-miR-802 | Cav1 |
| 753 | mmu-miR-340-3p | Serping1 | 1644 | mmu-miR-802 | Hltf |
| 754 | mmu-miR-340-3p | Rnf40 | 1645 | mmu-miR-802 | Dock4 |
| 755 | mmu-miR-340-3p | Scarf1 | 1646 | mmu-miR-802 | Arf4 |
| 756 | mmu-miR-340-3p | Pps | 1647 | mmu-miR-802 | Tmem110 |
| 757 | mmu-miR-340-3p | Ptafr | 1648 | mmu-miR-802 | Cyp51 |
| 758 | mmu-miR-340-3p | Blmh | 1649 | mmu-miR-802 | Pb1 |
| 759 | mmu-miR-340-3p | Emid2 | 1650 | mmu-miR-802 | Ttrap |
| 760 | mmu-miR-340-3p | Taok1 | 1651 | mmu-miR-802 | Klhdc1 |
| 761 | mmu-miR-340-3p | Gpatch3 | 1652 | mmu-miR-802 | Ube2m |
| 762 | mmu-miR-340-3p | Lin28 | 1653 | mmu-miR-802 | Mkln1 |
| 763 | mmu-miR-340-3p | Pdik1l | 1654 | mmu-miR-802 | Tspyl4 |
| 764 | mmu-miR-340-3p | Syf2 | 1655 | mmu-miR-802 | Pcdh18 |
| 765 | mmu-miR-340-3p | Cry2 | 1656 | mmu-miR-802 | Fli1 |
| 766 | mmu-miR-340-3p | E2f2 | 1657 | mmu-miR-802 | Cdc2l6 |
| 767 | mmu-miR-340-3p | Ttyh3 | 1658 | mmu-miR-802 | Pcdh21 |
| 768 | mmu-miR-340-3p | Ephb2 | 1659 | mmu-miR-802 | Car8 |
| 769 | mmu-miR-340-3p | Pex12 | 1660 | mmu-miR-802 | Psmd2 |
| 770 | mmu-miR-340-3p | Usp42 | 1661 | mmu-miR-802 | Gdf6 |
| 771 | mmu-miR-340-3p | Acaca | 1662 | mmu-miR-802 | Fbn2 |
| 772 | mmu-miR-340-3p | Arpc1a | 1663 | mmu-miR-802 | Pfn |
| 773 | mmu-miR-340-3p | Ggnbp2 | 1664 | mmu-miR-802 | Gch1 |
| 774 | mmu-miR-340-3p | 6330545A04Rik | 1665 | mmu-miR-802 | Dusp1 |
| 775 | mmu-miR-340-3p | Ypel2 | 1666 | mmu-miR-802 | Pcnx |
| 776 | mmu-miR-340-3p | Slc7a1 | 1667 | mmu-miR-802 | Cap2 |
| 777 | mmu-miR-340-3p | Trim25 | 1668 | mmu-miR-802 | Slc35f1 |
| 778 | mmu-miR-340-3p | Dgke | 1669 | mmu-miR-802 | Htr4 |
| 779 | mmu-miR-340-3p | Spag9 | 1670 | mmu-miR-802 | Nkrf |
| 780 | mmu-miR-340-3p | Hoxb3 | 1671 | mmu-miR-802 | Hspd1 |
| 781 | mmu-miR-340-3p | Icmt | 1672 | mmu-miR-802 | Rwdd4a |
| 782 | mmu-miR-340-3p | Chd5 | 1673 | mmu-miR-802 | Fut9 |
| 783 | mmu-miR-340-3p | Ajap1 | 1674 | mmu-miR-802 | Odz3 |
| 784 | mmu-miR-340-3p | P140 | 1675 | mmu-miR-802 | Aga |
| 785 | mmu-miR-340-3p | Mllt6 | 1676 | mmu-miR-802 | Sptlc1 |
| 786 | mmu-miR-340-3p | Pip4k2b | 1677 | mmu-miR-802 | Thoc2 |
| 787 | mmu-miR-340-3p | 3110001I20Rik | 1678 | mmu-miR-802 | Fga |
| 788 | mmu-miR-340-3p | Hdc | 1679 | mmu-miR-802 | Trak2 |
| 789 | mmu-miR-340-3p | Casc3 | 1680 | mmu-miR-802 | Ddx17 |
| 790 | mmu-miR-340-3p | Tgm3 | 1681 | mmu-miR-802 | Fkbp14 |
| 791 | mmu-miR-340-3p | Ptrf | 1682 | mmu-miR-802 | Ddx58 |
| 792 | mmu-miR-340-3p | Siglec1 | 1683 | mmu-miR-802 | Tmem25 |
| 793 | mmu-miR-340-3p | Plcb1 | 1684 | mmu-miR-802 | Ermp1 |
| 794 | mmu-miR-340-3p | Slc4a1 | 1685 | mmu-miR-802 | Pld3 |
| 795 | mmu-miR-340-3p | Bcl2l1 | 1686 | mmu-miR-802 | Atp6v1b2 |
| 796 | mmu-miR-340-3p | Chmp4b | 1687 | mmu-miR-802 | Fbl |
| 797 | mmu-miR-340-3p | Sox9 | 1688 | mmu-miR-802 | Nfatc1 |
| 798 | mmu-miR-340-3p | Sla2 | 1689 | mmu-miR-802 | Ddx26b |
| 799 | mmu-miR-340-3p | Rhbdf2 | 1690 | mmu-miR-802 | Agtpbp1 |
| 800 | mmu-miR-340-3p | Slc12a5 | 1691 | mmu-miR-802 | Atg2b |
| 801 | mmu-miR-340-3p | Ncoa5 | 1692 | mmu-miR-802 | Snf1lk2 |
| 802 | mmu-miR-340-3p | Ptpn1 | 1693 | mmu-miR-802 | Rcbtb1 |
| 803 | mmu-miR-340-3p | Tmepai | 1694 | mmu-miR-802 | Arpc2 |
| 804 | mmu-miR-340-3p | Zfp750 | 1695 | mmu-miR-802 | Cdc14b |
| 805 | mmu-miR-340-3p | Vapb | 1696 | mmu-miR-802 | Cdca4 |
| 806 | mmu-miR-340-3p | Ctsz | 1697 | mmu-miR-802 | Rhbdd1 |
| 807 | mmu-miR-340-3p | Ppp1r3d | 1698 | mmu-miR-802 | Stx17 |
| 808 | mmu-miR-340-3p | Frmd4a | 1699 | mmu-miR-802 | Rab43 |
| 809 | mmu-miR-340-3p | Il11ra1 | 1700 | mmu-miR-802 | Tshz3 |
| 810 | mmu-miR-340-3p | Gramd4 | 1701 | mmu-miR-802 | Arid2 |
| 811 | mmu-miR-340-3p | Dync1h1 | 1702 | mmu-miR-802 | Nolc1 |
| 812 | mmu-miR-340-3p | Celsr2 | 1703 | mmu-miR-802 | Stch |
| 813 | mmu-miR-340-3p | Ralgps1 | 1704 | mmu-miR-802 | Cct8 |
| 814 | mmu-miR-340-3p | Crim1 | 1705 | mmu-miR-802 | Aph1a |
| 815 | mmu-miR-340-3p | R3hdm2 | 1706 | mmu-miR-802 | Map2k1 |
| 816 | mmu-miR-340-3p | Snrk | 1707 | mmu-miR-802 | Scamp1 |
| 817 | mmu-miR-340-3p | Acss1 | 1708 | mmu-miR-802 | Pmpca |
| 818 | mmu-miR-340-3p | Agpat4 | 1709 | mmu-miR-802 | Pcdh8 |
| 819 | mmu-miR-340-3p | Zwint | 1710 | mmu-miR-802 | Slc5a3 |
| 820 | mmu-miR-340-3p | Rdh16 | 1711 | mmu-miR-802 | Arl8b |
| 821 | mmu-miR-340-3p | Iqce | 1712 | mmu-miR-802 | Cln5 |
| 822 | mmu-miR-374 | Rab10 | 1713 | mmu-miR-802 | Sox30 |
| 823 | mmu-miR-374 | Ncoa1 | 1714 | mmu-miR-802 | Erbb2ip |
| 824 | mmu-miR-374 | Itsn2 | 1715 | mmu-miR-802 | Nras |
| 825 | mmu-miR-374 | Mkx | 1716 | mmu-miR-802 | Slc16a1 |
| 826 | mmu-miR-374 | Vsnl1 | 1717 | mmu-miR-802 | Rnf12 |
| 827 | mmu-miR-374 | Larp5 | 1718 | mmu-miR-802 | Mpdz |
| 828 | mmu-miR-374 | Golph3 | 1719 | mmu-miR-802 | Ddx4 |
| 829 | mmu-miR-374 | Cwf19l2 | 1720 | mmu-miR-802 | Cept1 |
| 830 | mmu-miR-374 | Nr1d2 | 1721 | mmu-miR-802 | Snapc3 |
| 831 | mmu-miR-374 | Sema5a | 1722 | mmu-miR-802 | Cbfb |
| 832 | mmu-miR-374 | Vcpip1 | 1723 | mmu-miR-802 | Gnai3 |
| 833 | mmu-miR-374 | Abat | 1724 | mmu-miR-802 | Nup54 |
| 834 | mmu-miR-374 | Snx16 | 1725 | mmu-miR-802 | Ibtk |
| 835 | mmu-miR-374 | Rala | 1726 | mmu-miR-802 | Sntb2 |
| 836 | mmu-miR-374 | Ap3m1 | 1727 | mmu-miR-802 | Plekha6 |
| 837 | mmu-miR-374 | Ythdf3 | 1728 | mmu-miR-802 | Iqgap1 |
| 838 | mmu-miR-374 | Chordc1 | 1729 | mmu-miR-802 | Zfhx3 |
| 839 | mmu-miR-374 | Trpa1 | 1730 | mmu-miR-802 | Lum |
| 840 | mmu-miR-374 | Hivep2 | 1731 | mmu-miR-802 | Klhl12 |
| 841 | mmu-miR-374 | Capza2 | 1732 | mmu-miR-802 | AC122807.3 |
| 842 | mmu-miR-374 | Txlnb | 1733 | mmu-miR-802 | Mcart6 |
| 843 | mmu-miR-374 | Hltf | 1734 | mmu-miR-802 | Fbxw2 |
| 844 | mmu-miR-374 | Kif20a | 1735 | mmu-miR-802 | Nrk |
| 845 | mmu-miR-374 | Crispld1 | 1736 | mmu-miR-802 | Arsj |
| 846 | mmu-miR-374 | Cftr | 1737 | mmu-miR-802 | Pdcl |
| 847 | mmu-miR-374 | Nlgn1 | 1738 | mmu-miR-802 | Sfrs7 |
| 848 | mmu-miR-374 | Slc35d3 | 1739 | mmu-miR-802 | Tmem178 |
| 849 | mmu-miR-374 | Saps1 | 1740 | mmu-miR-802 | Kcng3 |
| 850 | mmu-miR-374 | Ppp1r3f | 1741 | mmu-miR-802 | Pik3r4 |
| 851 | mmu-miR-374 | Eif5a2 | 1742 | mmu-miR-802 | Asphd2 |
| 852 | mmu-miR-374 | Pde12 | 1743 | mmu-miR-802 | Rnf11 |
| 853 | mmu-miR-374 | Csmd1 | 1744 | mmu-miR-802 | Ermn |
| 854 | mmu-miR-374 | Rad21 | 1745 | mmu-miR-802 | Znhit6 |
| 855 | mmu-miR-374 | Tcf21 | 1746 | mmu-miR-802 | Slc4a10 |
| 856 | mmu-miR-374 | Wnt5a | 1747 | mmu-miR-802 | Sell |
| 857 | mmu-miR-374 | Slc25a21 | 1748 | mmu-miR-802 | Usp33 |
| 858 | mmu-miR-374 | Zhx1 | 1749 | mmu-miR-802 | Prkab1 |
| 859 | mmu-miR-374 | Atad2 | 1750 | mmu-miR-802 | Ankrd13c |
| 860 | mmu-miR-374 | Prkcd | 1751 | mmu-miR-802 | Setd2 |
| 861 | mmu-miR-374 | Vnn1 | 1752 | mmu-miR-802 | Foxj3 |
| 862 | mmu-miR-374 | Sec23a | 1753 | mmu-miR-802 | Zfp445 |
| 863 | mmu-miR-374 | Fancm | 1754 | mmu-miR-802 | Smtnl2 |
| 864 | mmu-miR-374 | Ube2h | 1755 | mmu-miR-802 | Slc30a1 |
| 865 | mmu-miR-374 | Gpr103 | 1756 | mmu-miR-802 | Pafah1b1 |
| 866 | mmu-miR-374 | Pb1 | 1757 | mmu-miR-802 | Khdrbs1 |
| 867 | mmu-miR-374 | Myst3 | 1758 | mmu-miR-802 | Timm22 |
| 868 | mmu-miR-374 | Rnf14 | 1759 | mmu-miR-802 | Mtch2 |
| 869 | mmu-miR-374 | Il2 | 1760 | mmu-miR-802 | Ckap5 |
| 870 | mmu-miR-374 | Anln | 1761 | mmu-miR-802 | Med13 |
| 871 | mmu-miR-374 | Tcerg1 | 1762 | mmu-miR-802 | Sqrdl |
| 872 | mmu-miR-374 | Bmper | 1763 | mmu-miR-802 | Ap4e1 |
| 873 | mmu-miR-374 | Dpy19l1 | 1764 | mmu-miR-802 | Acly |
| 874 | mmu-miR-374 | Arid4a | 1765 | mmu-miR-802 | Ptrf |
| 875 | mmu-miR-374 | Sema3e | 1766 | mmu-miR-802 | Bmp2 |
| 876 | mmu-miR-374 | Tacc1 | 1767 | mmu-miR-802 | Pak7 |
| 877 | mmu-miR-374 | Plk4 | 1768 | mmu-miR-802 | Flrt3 |
| 878 | mmu-miR-374 | Nt5dc1 | 1769 | mmu-miR-802 | Cd93 |
| 879 | mmu-miR-374 | Atp6ap2 | 1770 | mmu-miR-802 | Acss1 |
| 880 | mmu-miR-374 | Dmxl1 | 1771 | mmu-miR-802 | Ern1 |
| 881 | mmu-miR-374 | Ptpn12 | 1772 | mmu-miR-802 | Asxl1 |
| 882 | mmu-miR-374 | Foxo1 | 1773 | mmu-miR-802 | Apoh |
| 883 | mmu-miR-374 | Fgl2 | 1774 | mmu-miR-802 | Eif6 |
| 884 | mmu-miR-374 | Napepld | 1775 | mmu-miR-802 | Nfs1 |
| 885 | mmu-miR-374 | Nrn1 | 1776 | mmu-miR-802 | Sfrs6 |
| 886 | mmu-miR-374 | Rp2h | 1777 | mmu-miR-802 | Afmid |
| 887 | mmu-miR-374 | Srpk2 | 1778 | mmu-miR-802 | Sdc4 |
| 888 | mmu-miR-374 | Tm4sf4 | 1779 | mmu-miR-802 | Sec24c |
| 889 | mmu-miR-374 | Klhl7 | 1780 | mmu-miR-802 | Neto1 |
| 890 | mmu-miR-374 | Actn1 | 1781 | mmu-miR-802 | Vim |
| 891 | mmu-miR-374 | Col5a2 |  |  |  |
